# Genomic history of the origin and domestication of common bean unveils its closest sister species

**DOI:** 10.1186/s13059-017-1190-6

**Published:** 2017-03-29

**Authors:** Martha Rendón-Anaya, Josaphat M. Montero-Vargas, Soledad Saburido-Álvarez, Anna Vlasova, Salvador Capella-Gutierrez, José Juan Ordaz-Ortiz, O. Mario Aguilar, Rosana P. Vianello-Brondani, Marta Santalla, Luis Delaye, Toni Gabaldón, Paul Gepts, Robert Winkler, Roderic Guigó, Alfonso Delgado-Salinas, Alfredo Herrera-Estrella

**Affiliations:** 1 Laboratorio Nacional de Genómica para la Biodiversidad, Centro de Investigación y de Estudios Avanzados del IPN (Cinvestav), 36821 Irapuato, Guanajuato Mexico; 2 Departamento de Biotecnología y Bioquímica, Unidad Irapuato, Cinvestav, 36821 Irapuato, Guanajuato Mexico; 3 Bioinformatics and Genomics Programme, Centre for Genomic Regulation (CRG), Dr. Aiguader 88, 08003 Barcelona, Spain; 4 Universitat Pompeu Fabra (UPF), Dr. Aiguader 88, 08003 Barcelona, Spain; 5 Instituto de Biotecnología y Biología Molecular (IBBM), UNLP-CONICET, 1900 La Plata, Argentina; 6 EMBRAPA Rice and Beans, Biotechnology Laboratory, Santo Antônio de Goiás, GO 75375-000 Brazil; 7 Mision Biológica de Galicia (MBG)-National Spanish Research Council (CSIC), 36080 Pontevedra, Spain; 8 Departamento de Ingeniería Genética, Unidad Irapuato, Cinvestav, Irapuato, Guanajuato Mexico; 9 Department of Plant Sciences, University of California, Davis, CA 95616-8780 USA; 10 Departamento de Botánica, Instituto de Biología, Universidad Nacional Autónoma de México, 04510 Mexico City, Mexico

**Keywords:** Common bean, Domestication, Genomic introgression, Adaptive traits, Speciation

## Abstract

**Background:**

Modern civilization depends on only a few plant species for its nourishment. These crops were derived via several thousands of years of human selection that transformed wild ancestors into high-yielding domesticated descendants. Among cultivated plants, common bean (*Phaseolus vulgaris* L*.*) is the most important grain legume. Yet, our understanding of the origins and concurrent shaping of the genome of this crop plant is limited.

**Results:**

We sequenced the genomes of 29 accessions representing 12 *Phaseolus* species. Single nucleotide polymorphism-based phylogenomic analyses, using both the nuclear and chloroplast genomes, allowed us to detect a speciation event, a finding further supported by metabolite profiling. In addition, we identified ~1200 protein coding genes (PCGs) and ~100 long non-coding RNAs with domestication-associated haplotypes. Finally, we describe asymmetric introgression events occurring among common bean subpopulations in Mesoamerica and across hemispheres.

**Conclusions:**

We uncover an unpredicted speciation event in the tropical Andes that gave rise to a sibling species, formerly considered the “wild ancestor” of *P. vulgaris*, which diverged before the split of the Mesoamerican and Andean *P. vulgaris* gene pools. Further, we identify haplotypes strongly associated with genes underlying the emergence of domestication traits. Our findings also reveal the capacity of a predominantly autogamous plant to outcross and fix loci from different populations, even from distant species, which led to the acquisition by domesticated beans of adaptive traits from wild relatives. The occurrence of such adaptive introgressions should be exploited to accelerate breeding programs in the near future.

**Electronic supplementary material:**

The online version of this article (doi:10.1186/s13059-017-1190-6) contains supplementary material, which is available to authorized users.

## Background

The transition from hunting–gathering to agriculture is one of the major milestones in human evolution. An important, *sine qua non* consequence of this transition has been the domestication of crop plants and farm animals [1]. Furthermore, domestication provides an experimental model to study evolution in general, with several advantages, including the existence of ancestral populations, an established time frame (~10,000 years), and identifiable traits under selection for both domesticated plants and animals [2]. In this perspective, *Phaseolus* species are of particular interest because of the multiple domestications that have taken place in this genus. Indeed, of the 70–80 wild species that have been described, no less than five species have been domesticated in contrasting ecogeographic settings: common bean (*P. vulgaris* L*.*); lima bean (*P. lunatus* L.); runner bean (*P. coccineus* L.); tepary bean (*P. acutifolius* A. Gray); and year bean (*P. dumosus* Macfady). In addition, the first two species were independently domesticated at least twice—in Mesoamerica and in the Andes—implying that some domestication traits may have been selected multiple times, as shown by the determinacy trait in common bean [3]. This is in contrast with other crops that have been subjected to fewer domestication events, such as maize (single domestication [4, 5]) or rice and wheat (three domestications [6–9]). The multiple domestication phenomenon in *Phaseolus* provides an opportunity to examine to what extent similar selection pressures have led to convergent evolution at the molecular level [10]. Conversely, comparative genomics can illustrate the differential genetic control of adaptation to contrasting environments in which the different *Phaseolus* species were domesticated.

Although a New World origin of the genus has been established by phylogenetic studies [11], the geographic origin of *P. vulgaris* has been strongly debated. Initial evidence suggested the Peruvian–Ecuadorian region as the center of origin, given that accessions collected there have an ancient form of the seed storage protein phaseolin [12, 13]. However, based on an analysis of five loci, Bitocchi et al. [14] proposed that common bean originated in Mexico and then colonized the Southern hemisphere, giving rise separately to the Peruvian–Ecuadorian populations and the wild Andean gene pool, both phylogenetically derived from the Mesoamerican clade. Despite the uncertainty regarding the geographic origin of *P. vulgaris*, several lines of evidence from traditional (allozymes or seed proteins) and more recent molecular markers [15–17] converge in the establishment of two geographically and genetically isolated gene pools, one in Mesoamerica and one in the central to southern Andes. From these pools, two independent domestications took place starting ~8000 years ago [18–21], followed by local adaptations and further expansions. Accompanying these processes, not only has the genetic diversity of the domesticated varieties decreased due to domestication bottlenecks, but it would also appear that hybridization events between wild and domesticated populations have occurred through time, as suggested by morphological variation and microsatellite diversity [22–26], displacing the original genetic diversity in these regions [27, 28]. At the same time, however, introgressions from wild relatives may have permitted domesticated varieties to acquire adaptive traits. In this regard, gene flow has been crucial for the adaptation of maize cultivars to different environmental conditions [29], as well as for the introduction of morpho-agronomic traits that increase the commercial value of domesticated varieties of tomato [30]. Accordingly, gene flow also deserves careful examination in the context of common bean evolution.

This study aims at understanding how the current distribution of common bean was originated and how its genome has been shaped by domestication and through gene flow from its close relatives, to become the crop plant we know. Accordingly, we built a robust evolutionary model of common bean lineage divergence and domestication. We relied on the availability of two recently published annotated whole-genome sequences, of Mesoamerican [31] and Andean [32] origins, and re-sequenced ten additional accessions from Mesoamerica (MA) and three from the southern Andes (AN), together with five genotypes from the Peruvian–Ecuadorian area originating in the Amotape–Huancabamba Depression (AH), and 11 Mesoamerican *Phaseolus* species from the Vulgaris, Filiformis, Lunatus, Leptostachyus, Polystachios, and Tuerckheimii phylogenetic groups [11]. We focused on three essential, novel aspects of this model that are key to establishing the basic domestication pattern of common bean. First, a phylogenetic analysis of the presumed ancestral *P. vulgaris*, which has an extraordinarily broad distribution extending from northern Mexico to north-western Argentina, leads us to postulate that a cluster of wild populations in northern Peru–Ecuador actually represents a sibling species of *P. vulgaris*, which was not domesticated. Second, an analysis of the patterns of allelic admixture identifies signals of asymmetric intra-species and inter-species genomic introgression, which can represent the acquisition of adaptive traits by domesticated beans from its wild relatives. Third, we identify both shared and distinctive haplotypes associated with domestication traits between the Mesoamerican and Andean domestication processes.

## Results

### Genomic differentiation of *Phaseolus* species

Twenty-nine *Phaseolus* genomes, representing most of the species diversity in the genus, were sequenced at a coverage in the range of 8–20X (Additional file 1: Figure S1; Additional file 2: Tables S1 and S2). According to a previously proposed phylogenetic classification, which divides *Phaseolus* species into two sister clades [11], our sampling covered one of the three well-defined groups from clade A (Tuerckheimii) and had at least one representative species from each group of clade B (comprising all domestication events and having a broader distribution in the Americas), with an intentional bias towards the Vulgaris group. Raw reads were filtered and mapped against the *P. vulgaris* cv. BAT93 reference genome of 556.4 Mb (86% of the theoretical genome length of 650 Mb), as well as to a synteny-based pseudoassembly of BAT93 using the *P. vulgaris* G19833 genome as a scaffold. This pseudoassembly was produced using the SynMap tool at CoGe (https://genomevolution.org/coge/) with ≥ 4 contiguous syntenic CDSs between assembled tracks in order to construct longer chromosomes with more certainty of the order and sense of the scaffolds than in the current BAT93 genome version. The calculated breadths of coverage (number of bases of the reference genome that were covered during the mapping process) were congruent with the phylogenetic closeness of each accession to *P. vulgaris* BAT93 [11], as they were in the range of 56–77% for species from the Filiformis, Lunatus, Leptostachyus, Polystachios, and Tuerckheimii groups; 60–88% for species from the Vulgaris group (e.g. *P. acutifolius* or *P. coccineus*), and up to > 90% for most *P. vulgaris* accessions (Additional file 2: Table S2).

**Fig. 1:**
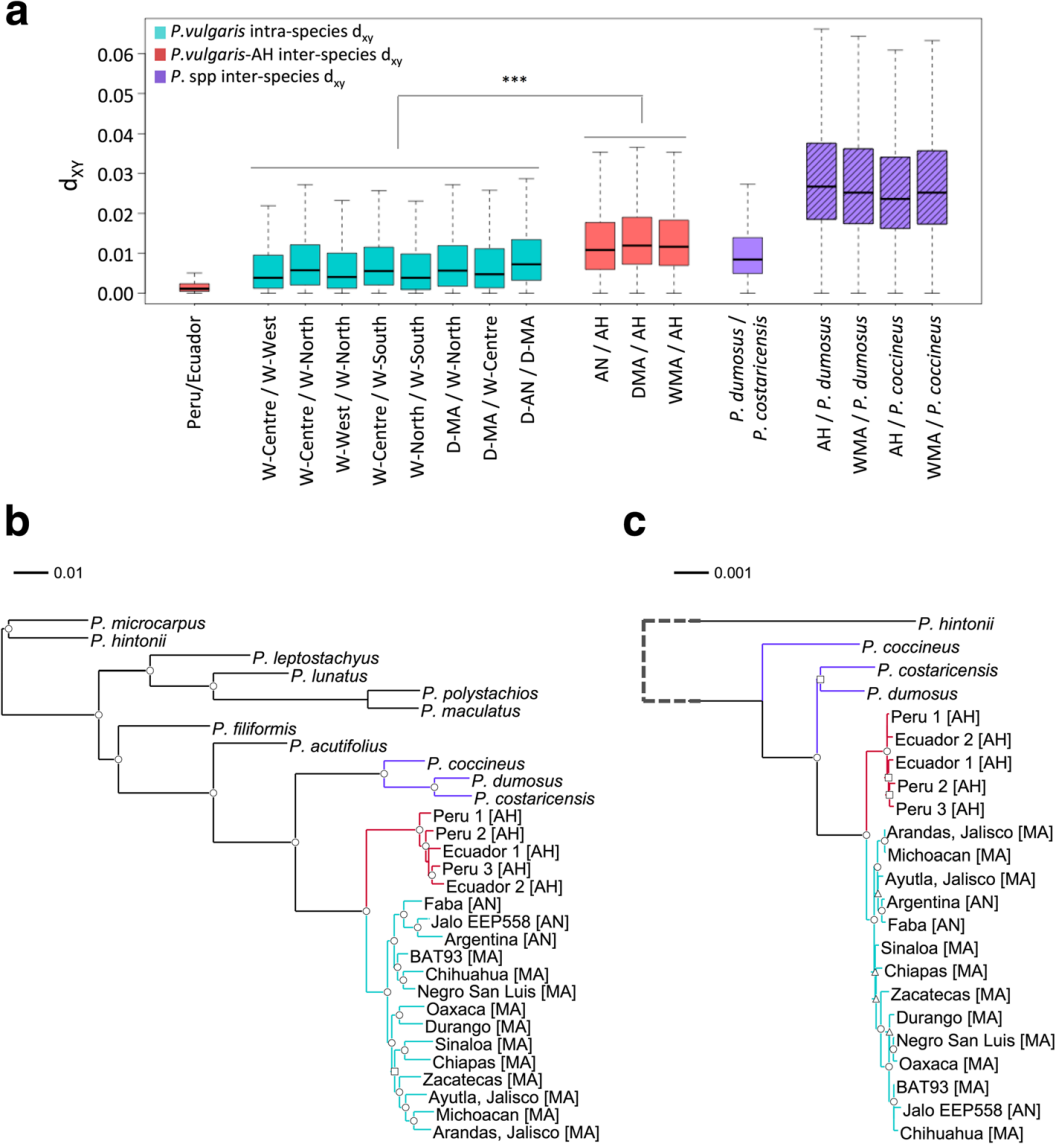
Species definition within the Vulgaris group according to their phylogenomic profile. **a** Absolute genetic divergence between *Phaseolus* subpopulations, showing inter-species and intra-species divergence comparisons. The difference of d_XY_ values (Kruskal-Wallis *p* value = 0.014) calculated within *P. vulgaris* subpopulations and between *P. vulgaris* and the AH subpopulation, is highlighted with (***). **b** ML tree with non-parametric SH branch support based on 460,000 single nucleotide polymorphisms randomly chosen across the genome. **c** ML tree with non-parametric SH branch support based on 55 Kb of the chloroplast genome. The long branch length separating *P. hintonii* from the Vulgaris species is graphically represented with a dotted line. Branch support: SH-aLRT = [0.75;0.85], triangles; SH-aLRT = [0.85;0.95], squares; SH-aLRT > 0.95, circles. In both tree topologies and the box plot, *P. vulgaris* accessions are highlighted in cyan, *P. pseudovulgaris* in red and *Phaseolus* species from the Vulgaris groups in purple

Based on the collection sites of the *P. vulgaris* accessions, we grouped them in subpopulations (Table 1) that became useful for calculations requiring allele frequencies, such as intra-species and inter-species pairwise absolute genetic divergences (d_XY_, calculated on 5 Kb non-overlapping windows; [33]) (Fig. 1a). We observed *P. vulgaris* intra-species average divergence values below 0.009 and higher d_XY_ values between *P. vulgaris* and its sister species *P. coccineus*, *P. dumosus*, and *P. costaricensis* Freytag & Debouck, in the range of 0.026–0.03. From this analysis, two contrasting results were noteworthy. First, accessions from the AH zone belonged to a narrowly restricted population, both at the geographic and genetic levels, as they were the least divergent accessions among all comparisons (d_XY_ = 0.0023). Second, the AH subpopulation and any other *P. vulgaris* group were equally divergent (d_XY_ ≈ 0.014), as were the two well-defined sister species, *P. dumosus* and *P. costaricensis* (d_XY_ = 0.011). Not only were the d_XY_ values within *P. vulgaris* subpopulations and between *P. vulgaris* and AH accessions different (Kruskal-Wallis *p* value = 0.014), but the comparison of inter-species and intra-species distances (Fig. 1a) indicated that they were all derived from independent populations (Kruskall Wallis *p* value = 0.007). The genome-wide d_XY_ calculation indicates that the AH subpopulations represent a different lineage, divergent enough from *P. vulgaris* and from other close members of the Vulgaris group (*P. dumosus*, *P. costaricensis*, or *P. coccineus*) to be considered a different lineage.

**Table 1:** *Phaseolus* subpopulations, grouped based on their sites of collection

Subpopulation	Accessions	Origin	Type
North	Sinaloa; Durango	Mesoamerican	Wild
Center	Zacatecas; Jalisco	Mesoamerican	Wild
South	Chiapas; Oaxaca	Mesoamerican	Wild
West	Michoacán; Jalisco	Mesoamerican	Wild
DMA	BAT93; Negro San Luis; Chihuahua	Mesoamerican	Domesticated
AN	Jalo EEP558; Faba Andecha; G19901	Andean	Domesticated + Wild
AH	G21244; G21245; G23587; G23724; G23582	Amotape–Huancabamba Zone in Peru/Ecuador	Wild

### Uncovering the closest sister clade of *P. vulgaris*

We reconstructed the phylogenetic relationships of the species using genome-wide detected single nucleotide polymorphisms (SNPs) (17.2 e^6^ SNPs were detected, and filtered to remove unique polymorphisms to a total of 7.4e^6^; Additional file 2: Table S3). This analysis uncovered an unpredicted novel lineage within the Vulgaris group (Fig. 1b). In contrast to previous reports in which wild accessions from northern Peru and Ecuador formed a clade derived from MA wild subpopulations [14], our maximum-likelihood tree (produced with PhyML using aLRT non-parametric SH branch support [34]) placed these individuals in a separate clade (SH-aLRT = 1), sister to all *P. vulgaris* genotypes, Andean and Mesoamerican. This signal remained consistent in each linkage group when individual phylogenies were reconstructed using specific SNPs for each chromosome (6.1e^5^ SNPs on average per chromosome; Additional file 1: Figures S2–S4). We further corroborated this evolutionary relationship using a sequenced 55-Kb chloroplast genome fragment (cpDNA). The phylogenetic signal resulting from the cpDNA (Fig. 1c) was consistent with that observed for nuclear markers (Fig. 1b), pointing to a divergence of the AH genotypes and the *P. vulgaris* lineage that predated the split of the MA and AN wild gene pools. Moreover, these results confirm the hybrid speciation between *P. vulgaris* (maternal contributor) and *P. coccineus* (paternal contributor) that gave rise to *P. dumosus* [35, 36]. The high nuclear affinity between *P. dumosus* and *P. coccineus* and the remarkable chloroplast closeness of *P. dumosus* and *P. vulgaris* indicates that the cpDNA is indeed telling a linear evolutionary history that does not seem to be strongly influenced by recombination events in the organelle.

To provide a temporal frame of the divergence of the AH/*P. vulgaris* lineages, coalescent simulations with an uncorrelated lognormal relaxed molecular clock were performed using both nuclear (two independent sets of 150 and 170 genes) and plastid markers (55 Kb of the chloroplast genome). Several time priors were tested according to previous reports [11, 32], e.g. 0.165 Mya of divergence between the MA and AN *P. vulgaris* gene pools (Additional file 3: Tables S4 and S5); clock rates and μ values were adjusted for each dataset according to the calculated pairwise absolute genetic divergences (Additional file 3: Tables S4 and S5). Our results corroborated an early split of the AH lineage: the chloroplast genome shows a divergence time of 0.9 My [0.5–1.4, 95% Highest Posterior Density interval ( HPD)] while the nuclear markers show a divergence time of 0.26 My (0.02–0.7, 95% HPD), much earlier than the separation of the MA and AN gene pools (0.2 My with 0.07–0.3 95% HPD for the plastid markers; 0.002 My with 1.5E-4–5.9E-3 95% HPD for the nuclear gene sets (Additional file 3: Tables S6–S11; Figures S5–S10). The discrepancies observed in the coalescent results (older divergence using cpDNA than nuclear DNA) are most likely attributable to nuclear recombination events, as has been documented in other plant models [37–39].

Altogether, these results provide further support to our phylogenomic inferences and indicate that the AH group should be considered a separate lineage within the Vulgaris group. Based on these findings and the following data, we henceforth denote the AH group as “*Phaseolus pseudovulgaris*.”

### Metabolomic profiling differentiates *Phaseolus* species and implicates flavonoid production as a phenomenon accompanying species radiation

**Fig. 2:**
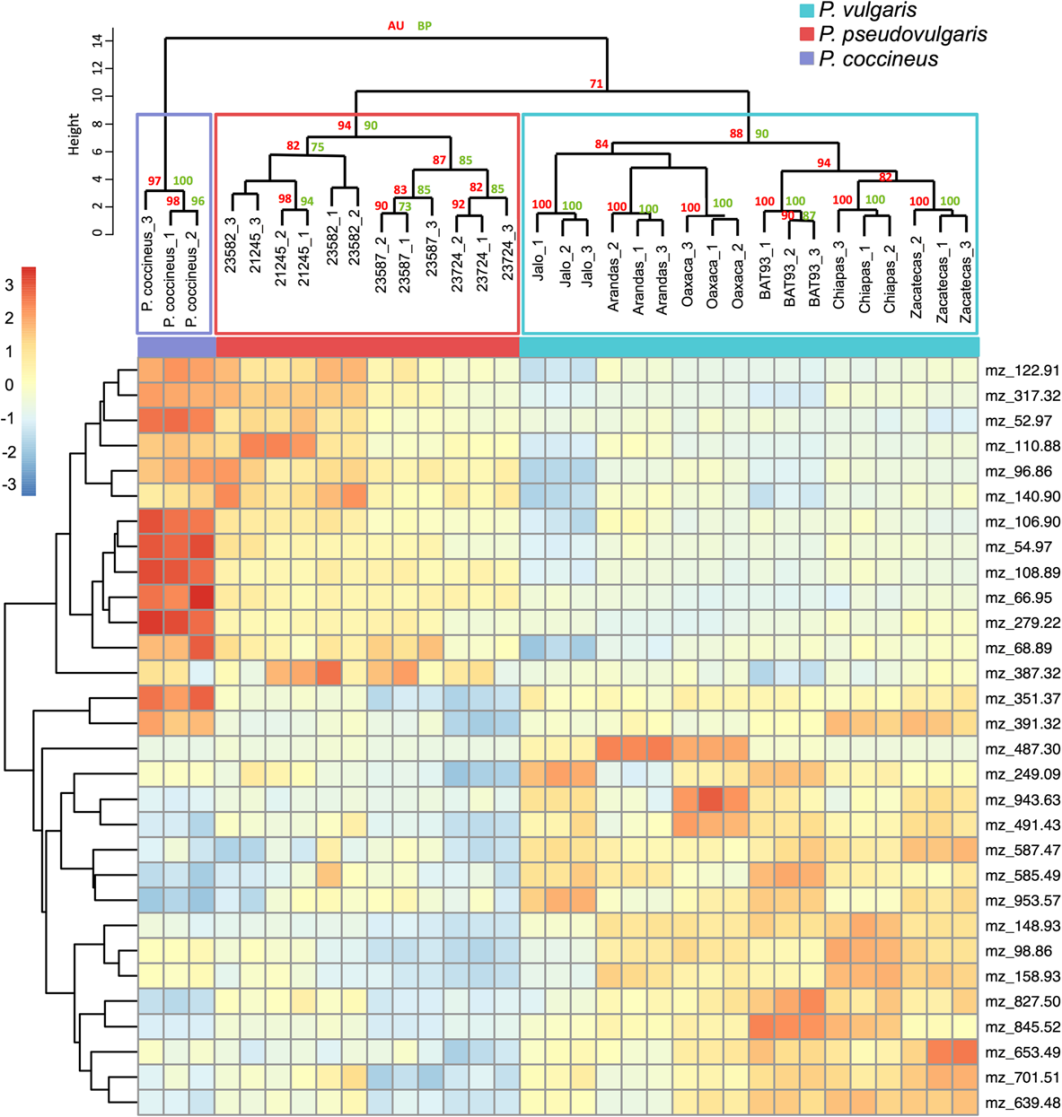
Metabolomic profiles of *Phaseolus* species. The *heatmap* shows the 30 most informative mass signals from extracts of young trifoliate leaves that explain inter-species differences between *P. vulgaris*, *P. pseudovulgaris*, and *P. coccineus*. The associated horizontal dendrogram reproduces the phylogeny of the accessions, while the vertical dendrogram clusters mass signals according to their abundance. Approximately unbiased probabilities (AU) and bootstrap support (BP) ≥ 70 are displayed in the horizontal dendrogram

Similar morphology appears to have hidden evolutionary relationships between *Phaseolus* lineages, particularly when *P. vulgaris* and *P. pseudovulgaris* are contrasted. To establish a phenotypic discrimination of the species, other than morphological traits, we used high-throughput, non-targeted mass fingerprinting [40, 41]. Combining direct-injection electrospray mass spectrometry (DIESI-MS) and hierarchical cluster analysis (HCA), we constructed metabolic profiles of *P. vulgaris*, *P. pseudovulgaris*, and *P. coccineus* accessions from young trifoliate leaves (Fig. 2). More than 1000 different mass to charge signals (*m*/*z*) were recovered, representing the “metabolic space” of each accession. After mass error removal and signal filtering, 318 high quality mass signals of metabolites were kept for further analyses. HCA of the 100 most abundant metabolites correctly isolated *P. coccineus* as the outgroup and discriminated *P. vulgaris* accessions into wild or domesticated types. More importantly, the *P. vulgaris* accessions were separated from their sibling species, placing these accessions in two independent clades (Additional file 1: Figures S11 and S12). Using a data mining approach [42], we identified the 30 variables that best explained the metabolic differences between the common bean populations. The dendrogram constructed from those variables replicated the phylogeny described in the previous section, with bootstrap and approximately unbiased (AU) probabilities supporting the topology (Fig. 2).

Using high-resolution liquid chromatography–mass spectrometry (LC-MS) data, we identified 44 metabolites, 25% of which were among the 100 variables that best explained inter-species differences. Most of the metabolite diversity in this set corresponded to flavonoids, such as the isobars of luteolin and kaempferol, or the coumarin derivative 4-methylumberlliferone (Additional file 2: Table S12) that play crucial roles during legume-microbe interactions in the rhizosphere (reviewed by [43]).

### Asymmetric intra-species and inter-species genomic flow in Mesoamerica and across hemispheres

In spite of its preferential autogamy, *P. vulgaris* cannot be considered to have a closed reproductive system, as it maintains outcrossing rates in the range of 1–70%, depending upon the experimental conditions [44, 45]. Therefore, combining the dynamic estimator of the degree of introgression between subpopulations (f_d_, a modified version of Patterson’s D statistic) and the absolute genetic divergence (d_XY_) [46–48], we looked for allelic admixture signals within and between *Phaseolus* species. Several triads (P_1_P_2_P_3_O) were considered to estimate the f_d_ parameter, permuting the donor (P_3_) and receptor (P_1_,P_2_) subpopulations (Table 1) and fixing *P. hintonii* as the outroup (O). We defined introgressed blocks as those windows that belong to the top 5% f_d_ outliers and, at the same time, display d_XY_ values smaller than the mean d_XY_ across the whole genome. Genomic windows displaying such traits were condensed into larger blocks that, in several cases, were close to the size of recombination units.

**Fig. 3:**
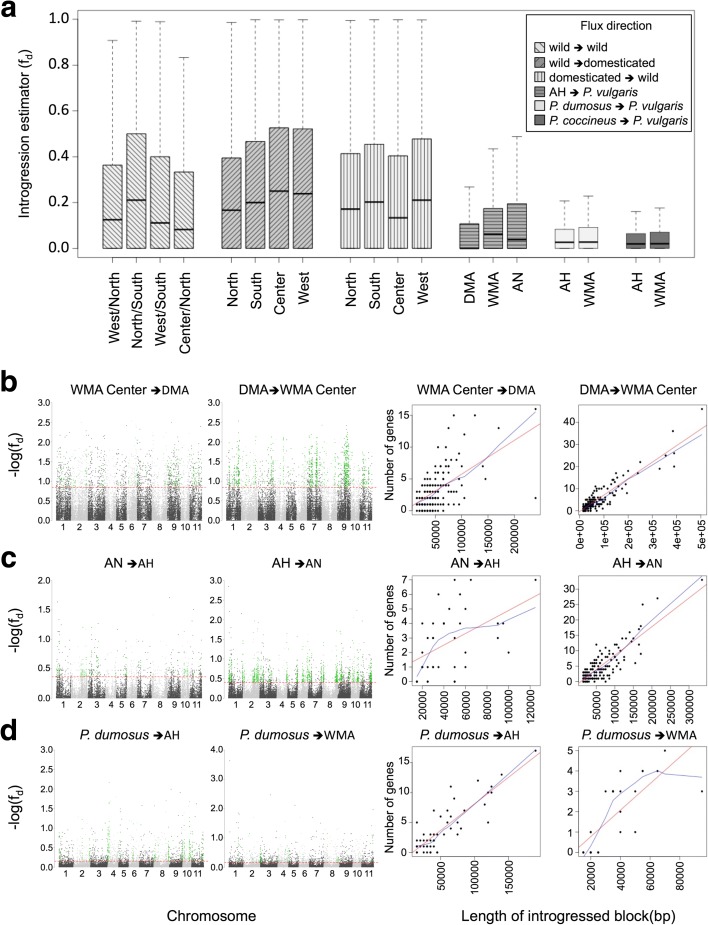
Introgression rate (f_d_) depends on phylogenetic distance between subpopulations. **a** Global f_d_ estimations for different triads of *Phaseolus* subpopulations. **b**–**d** Introgression signal across the linkage groups divided into 5-Kb non-overlapping windows is represented in *Manhattan plots* (*left panels*); the *red threshold lines* show the top 5% f_d_ outliers in each comparison, and strong signals of introgression (f_d_ + d_XY_) are highlighted in *green*. The number of genes encoded in each introgressed block is represented in *scatterplots* (*right panels* – *colored lines*: linear [*red*] and local [*blue*] regressions). In (**d**), the donor subpopulation is conformed by *P. dumosus* and *P. costaricensis*

**Fig. 4:**
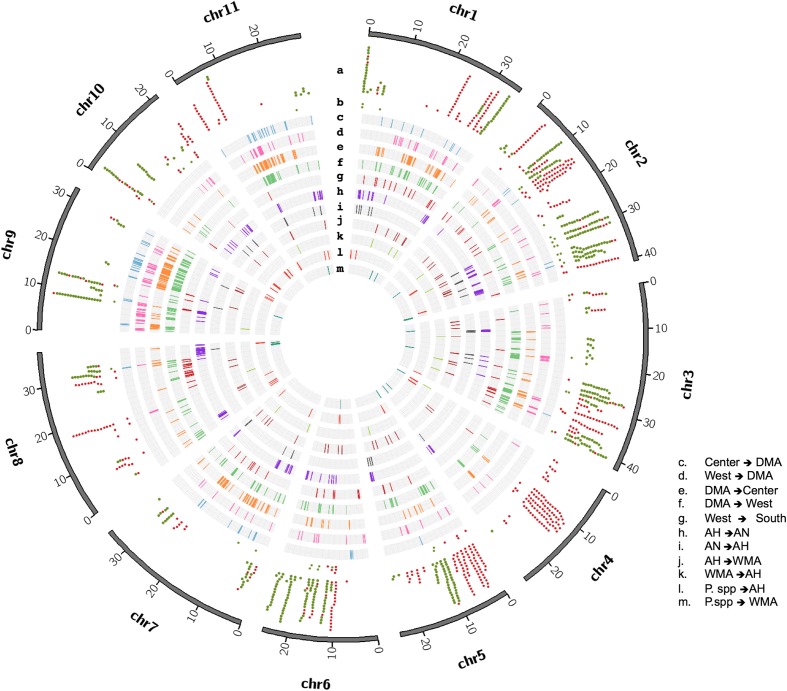
Introgression and domestication signals across *P. vulgaris* linkage groups. **a** Domestication genes; *green*: common to both COD; *red*: MA-specific. **b** lncRNAs domestication haplotypes (same colors as (**a**)). **c**–**k** Introgressed blocks: (**c**, **d**) wild ⇐ domesticated; (**e**, **f**) domesticated ⇐ wild; (**g**) wild ⇐ wild; (**h**–**k**) AH ⇐ *P. vulgaris*; (**l**, **m**) *P. dumosus/P. costaricenses* ⇐ *P. vulgaris*

We observed a clear tendency of increased introgression signals as we compared phylogenetically closer subpopulations. That is, intraspecific introgression occurred with higher frequency than interspecific introgression (Figs. 3a and 4c–m). The f_d_ values were close to 0.3 between *P. vulgaris* subpopulations, regardless of their wild or domesticated origin, whereas inter-species f_d_ values dropped to 0.05–0.1. While admixture can occur in both directions, we observed a larger genomic contribution in terms of the total length of introgressed tracks and transferred protein coding genes (PCGs) from domesticated into wild subpopulations (5.7–17.1 Mb) than from wild into domesticated genotypes (4.1–8.2 Mb; Additional file 1: Figures S13–S16). This was particularly evident when we took the Central subpopulation (Zacatecas and Jalisco) of wild MA genotypes as the receptor in the triad (Fig. 3b), which is consistent with local records that place Zacatecas and Jalisco among the most important states that produce common bean in Mexico and the widespread distribution of wild *P. vulgaris*, especially in Jalisco [49].

Interestingly, we detected asymmetric gene flow between the Northern and Southern American hemispheres, taking the AH genotypes as an intermediate subpopulation (Additional file 1: Figure S17). Although introgression was detected in both directions, from AH to MA and from AH to AN, it was stronger towards the Andean accessions (Fig. 3c), a fact that could be explained by the geographic closeness of the populations along the Andean corridor (e.g. [50]) and the lower levels of genetic diversity in the southern Andean region [14] that contribute to the maintenance of long introgressed blocks. Furthermore, the AH subpopulation appeared to be preferentially autogamous based on two observations: first, genetic diversity in the Amotape–Huancabamba Depression group was lower than in any other tested subpopulation (π_AH_ = 1.7e^–3^, π_South_ = 5.7e^–3^, π_West_ = 3.3e^–3^, π_North_ = 6.6e^–3^, π_Center_ = 5.4e^–3^, π_DMA_ = 4.3e^–3^, π_AN_ = 6.1e^–3^; Additional file 1: Fig. S18); second, introgression signals were weaker when these genotypes were permuted as receptors in the test triad (Fig. 3c). These results indicate that while *P. vulgaris* plants growing in the Southern hemisphere could be cross-pollinated by their AH wild neighbors, this did not occur generally in the opposite direction. It is also possible that interspecific introgressions from sister species that reached the Southern hemisphere, such as *P. coccineus* or *P. dumosus* (Fig. 3d), enhanced the differentiation of *P. pseudovulgaris* from *P. vulgaris* (Additional file 1: Figure S19).

### Parallel domestication events share signatures of selection in both common bean gene pools

An advantage of our approach was that whole-genome re-sequencing of individuals allowed us to define haplotypes across linkage groups and test their association with domesticated phenotypes in a case-control design. We looked for haplotype clusters (i.e. clustering of haplotypes on a localized basis: at the position of each genetic marker, haplotypes are clustered according to their similarity in the vicinity of the position) in 19 accessions including all *P. vulgaris* and *P. pseudovulgaris*. Two combinations of phenotypes (cases) were evaluated, one including the three domesticated accessions from MA and the second adding the two AN domesticated cultivars to define those haplotypes common to both domestication processes; the rest of the accessions were kept as controls. To test the haplotype–phenotype association, we followed the two-layer hidden Markov model with linear approximation implemented in hapQTL [51]. Haplotypes strongly associated with the domestication process were defined as such if their calculated Bayes factors were higher than those obtained after permuting case-control labels. The effect of such haplotypes (e.g. altering coding sequences, 5’/3’ UTRs or introns) was further evaluated. We selected as domestication candidates those genes that contained at least two markers with high association factors that were affecting regulatory regions, had non-synonymous effects on the coding sequence, or altered splicing sites or stop codons.

Following this procedure, we identified 599 genes with haplotypes shared between domesticated genotypes from MA and AN, and 628 genes with haplotypes specific to MA domesticated accessions (Fig. 4a; Additional file 4: Table S13; Additional file 5: Table S14). Similarly, 52 and 45 long non-coding RNAs (lncRNAs) with domestication-associated haplotypes were shared by the two centers of domestication (CODs) and within MA, respectively (Fig. 4b). These observations indicate that domestication has affected PCGs and regulatory elements, whose functions and targets should be further explored.

### Differential shaping of the common bean genome by domestication and genomic introgression

**Fig. 5:**
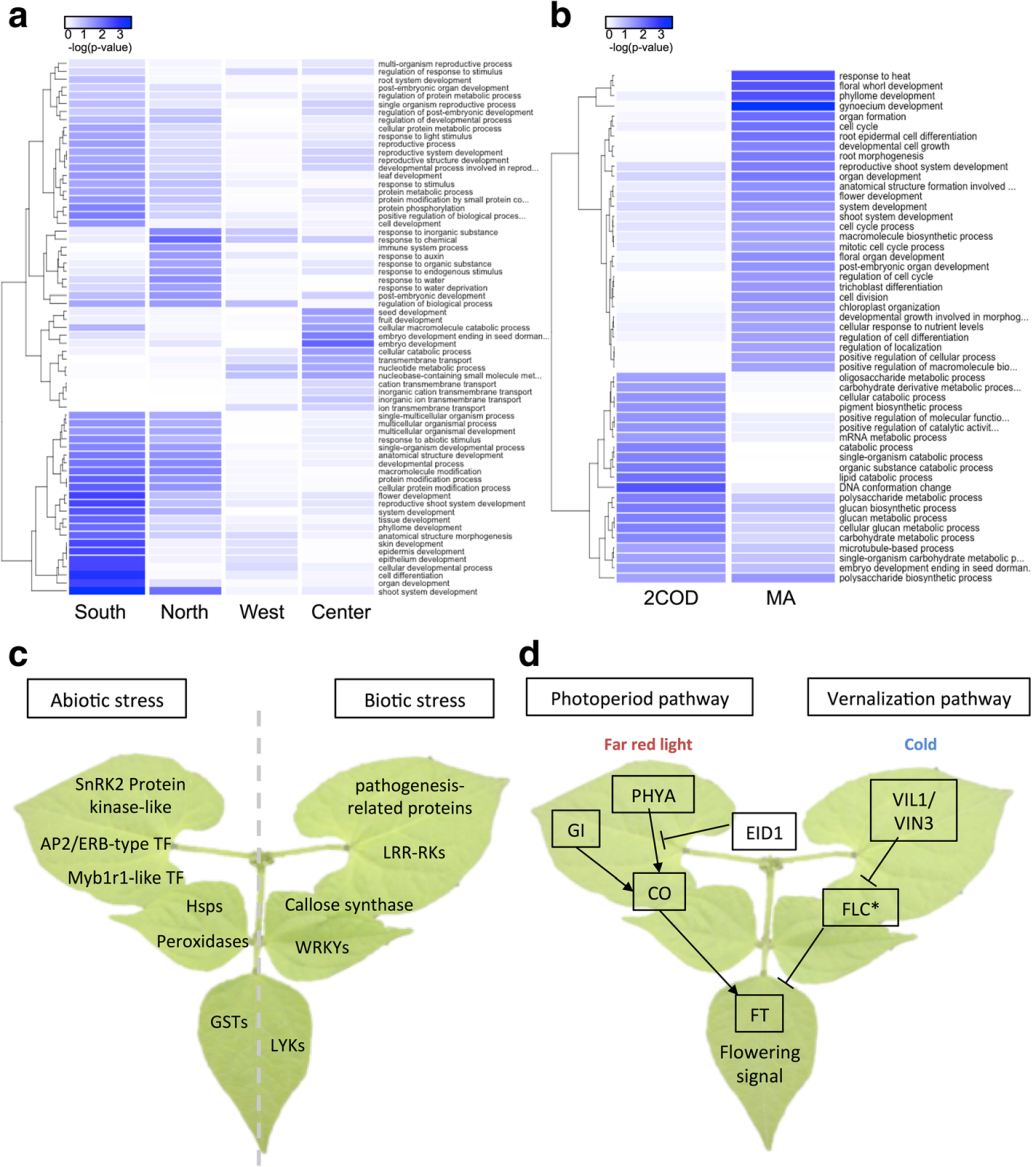
Functional description of domestication vs. introgression genes and pathways. *Heatmaps* show GO enrichments from genes within genomic blocks introgressed from wild MA subpopulations into domesticated MA accessions (**a**) or with domestication-associated haplotypes (**b**). **c** Examples of stress response genes that were mobilized by hybridization events from wild into domesticated individuals. **d** Photoperiod sensitivity and vernalization pathways, which confer a key domestication trait, are depicted. All genes except for those marked with an asterisk (*) share haplotypes in both centers of domestication that differentiate them from wild individuals

The functional descriptions of PCGs transferred by introgression events revealed several pathways of potential importance for crop improvement. First, GO terms related to cell wall biogenesis and organization, and pectin and cell wall polysaccharide metabolic processes, were enriched among introgressed genes transferred from *P. coccineus* and *P. dumosus/P. costaricensis* into *P. vulgaris*, which could have contributed to the acquisition of pathogen resistance in *P. vulgaris* [52]. Enrichment of functional terms associated with hormone-mediated signaling pathways, reproductive processes, post-embryonic development, and the formation of reproductive organs was associated with gene flow among *P. vulgaris* subpopulations. Contrary to the mobility of genes behind reproductive processes within *P. vulgaris* subpopulations, such categories were not statistically enriched when *P. pseudovulgaris* and *P. vulgaris* were evaluated. Interestingly, as reported in other crops [53], genes involved in biotic and abiotic stress responses were transferred in most of the *P. vulgaris* triads in both directions (Additional file 1: Figures S20 and S21; Additional file 6: Tables S15; Additional file 7: Table S16; Additional file 8: Table S17), implying that the continuous movement of such loci favored the adaptation of common bean to different habitats. Genes within these categories corresponded to WRKYs, leucine-rich repeat receptor kinases, and pathogenesis-related proteins, among others (Fig. 5a and c).

Screening of protein definitions associated with the domestication gene candidates identified 21 disease resistance genes and several significantly enriched GO categories (Fisher’s exact test, *p* < 0.05) that could be easily linked to the emergence of domestication traits (Fig. 5b; Additional file 9: Table S18; Additional file 10: Table S19). For instance, haplotypes common to both CODs affected components of the sucrose/starch biosynthetic pathway (directly related to starch content in the seeds), the regulation of reproductive processes (involving homologs of transcription factors such as WOX2 for embryonic patterning, or GTE1, which promotes seed germination), inflorescence development and meristem determinacy (e.g. homologs of the homeobox gene *KNOTTED1*). In addition, we identified genes particularly marked by SNPs in their regulatory regions, such as a homolog of *NODULATION-SIGNALLING PATHWAY 2* (*NSP2*), which is involved in Nod factor signaling in legumes, and several genes related to the dormancy and photoperiod sensitivity pathways (Fig. 5d). Other enriched categories such as chromatin assembly, nucleosome organization, and the regulation of histone methylation, were suggestive of epigenetic control in the emergence of domestication traits, which should be explored further. Among genes with MA haplotypes, we identified enriched GO categories particularly related to the development of reproductive structures or other organ formation (including homologs of transcription factors KAN2 or AS1), and genes directly involved in auxin transport and homeostasis or nodulation (*EARLY NODULIN 93*).

## Discussion

### *P. pseudovulgaris* emerged by allopatric speciation in the Amotape–Huancabamba Depression before the split of both *P. vulgaris* gene pools

**Fig. 6:**
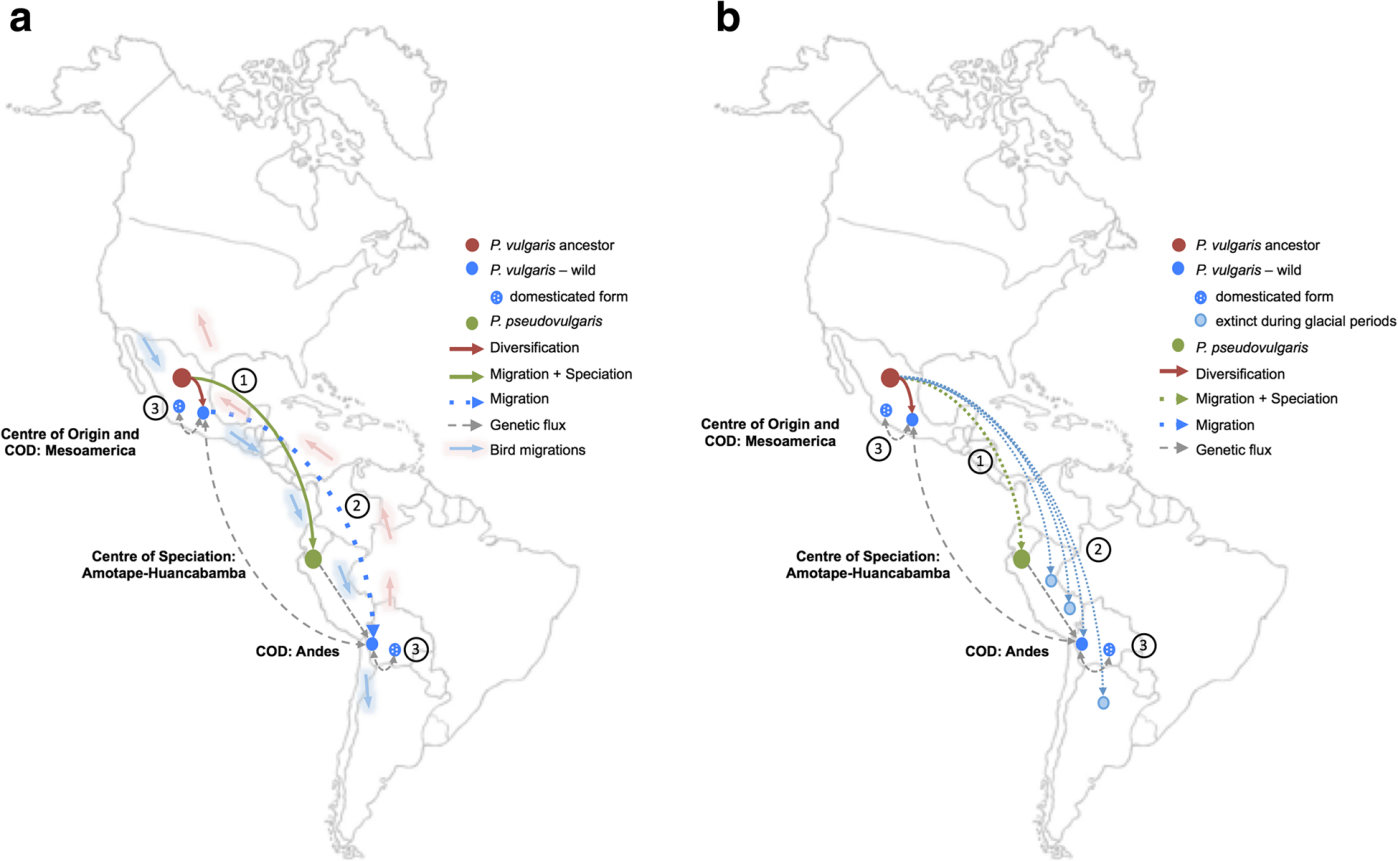
Spatio-temporal models of common bean migrations and lineage divergence in America. **a** Two-waved model of migration mediated by bird migrations. **b** Diversity extinction in the Southern hemisphere caused by glacial periods. Under both models, migration from the MA to AH region, followed by speciation (1) predates the split of *P. vulgaris* lineages (2); domestication corresponds to the most recent evolutionary event (3)

Our combined genomic and phenotypic data support the Mesoamerican origin of common bean but focus further questions on the diversification steps immediately before and after *P. vulgaris* speciation. We supply strong evidence pointing to an early speciation event in the western tropical Andes, which clarifies most of the discrepancies introduced by noisy phylogenetic signals of genotypes collected in the northern Peru–Ecuador area of Amotape–Huancabamba [14]. Indeed, this enclosed area has been described as a transition zone between the Northern and Central Andes, where climate dynamics and oro-geographic conditions have produced the highest degree of plant species diversity and endemism along the Andean Mountains [54–56]. Furthermore, the AH region represents a contrasting environment compared with that of other wild *P. vulgaris* in the Andes, including populations in Colombia and Venezuela (Mesoamerican gene pool [57]) and those in central and southern Peru, Bolivia, and Argentina (Andean gene pool [50]). Following this line of evidence, we propose a two-waved migration event in which *P. vulgaris*—or an ancestral form of the species—dispersed from Mesoamerica, reaching the AH zone between Northern and Central Andes, where it remained isolated and underwent allopatric speciation (Fig. 6a). This could have occurred through seed dispersal by birds [57, 58] following migration routes along the narrow isthmus connecting North and South America and following the Andean corridor of mountains but not necessarily reaching the western side of Peru and Ecuador when migrating from south to north [59]. Hundreds of thousands of years later, a small population of *P. vulgaris* with Mesoamerican genetic background likely invaded the Central and Southern Andes, giving rise to the second gene pool that was later domesticated. A second spatiotemporal model considers glacial periods in the Southern Andes during the Pleistocene (Fig. 6b). The fact that other *Phaseolus* species from the Vulgaris group can be found in Central America, reaching Colombia, suggests that seeds from this group, including the ancestral lineage of *P. vulgaris*, spread into South America across the Isthmus of Panama or by long-distance dispersal waves that have been dated for terrestrial organisms at 20 Mya and 6 Mya [60]. Climatic changes during the Pleistocene resulted in recurrent expansions and contractions of *Phaseolus* populations, placing them in glacial refuges and limiting gene flow between them. The Amotape–Huancabamba Depression could have been an important glacial refuge, favoring the isolation and diversification of the AH populations around 1 Mya, which coincides with our cpDNA coalescent results. The outcome of a glacial period spanning 140–180 Kya (reviewed by [61]) could have been a small remaining founder population of *P. vulgaris* in South America that was domesticated afterwards, as this glacial period matches the age of the split between the MA and AN gene pools and the suggested bottleneck duration in the Andean wild population [32].

In terms of phenotypic distinctiveness, we identified secondary metabolites such as flavonoids as variables that allow inter-species discrimination. It is noteworthy that among them we found luteolin, a strong inducer of Nod gene expression [62, 63], a chemo-attractant and a growth regulator of rhizobia [64]; kaempferol, a flavonol involved in the regulation of auxin transport in response to rhizobia [65]; and 4-methylumbelliferone, implicated in controlling lateral root formation [66]. In this regard, *Phaseolus* species have the capacity to select their symbionts from coexisting soil bacteria [67, 68]. A survey of nodule bacteria [69] revealed a clear preference for nodulation by *Bradyrhizobium* in most *Phaseolus* species and a shift to *Rhizobium* nodulation in the Vulgaris clade. These observations suggest that alterations in legume-root nodule symbiosis and symbiont preference shifts have accompanied *Phaseolus* speciation and diversification in the Americas.

### Insights into genetic barriers for reproduction in the genus

Given the short time period separating the AH *Phaseolus* populations from their *P. vulgaris* relatives from MA and AN, reproductive barriers have not been fully established, as reflected by introgression signatures from AH into Andean genotypes (Fig. 3) and the observation of weedy populations in the AH Zone [70]. This could be attributable to the morphological similarity between species (Additional file 2: Table S20). At the same time, the limited outcrossing of MA genotypes with AH, but not necessarily between AH and AN genotypes, could be due to the geographic barriers imposed by the Andean corridor; however, this does not exclude the possibility of outcrossing between the AH subpopulations and neighboring *P. vulgaris* from Venezuela and Colombia. The selective gene flow to the AN genotypes is also in agreement with a previous report of unsuccessful crosses between a genotype from Cajamarca, Peru (G21245), included in our sampling, and two tester lines from MA (G04830) and AN (G00122) [71]. In the same report, crosses of other genotypes showed hybrid weakness; accession G21245 was successfully crossed with 36% and 75% of the tested MA and AN genotypes, respectively, showing an asymmetric reproductive barrier [71].

The lack of an introgression signal affecting loci implicated in reproductive processes between *P. vulgaris* and *P. pseudovulgaris* gives further support to the reproductive isolation of accessions endemic to the AH Zone. Alleles that determine species phenotypes tend to introgress at a very low frequency [72]. Therefore, the observation that genes involved in reproductive processes are not transferred strongly suggests that these loci are important for the establishment of reproductive barriers between these close species. Previous studies have attempted to identify the genetic sources of incompatibility between the AN and MA gene pools, so far attributable to the root-expressed and shoot-expressed semi-dominant alleles DOSAGE-DEPENDENT LETHAL 1 (*DL1*) and *DL2* [73]. The differential introgression that we observe within *P. vulgaris* and between *P. vulgaris* and *P. pseudovulgaris* might provide insight into the genetic basis of the reproductive isolation in the genus.

### Modern cultivars result from the combined outcome of domestication and adaptive introgressions

Standing genetic diversity is a prerequisite for more rapid adaptation in response to selection pressures and constitutes the raw material to develop improved breeds or cultivars [74]. A survey of the use of wild germplasm in crop improvements over the last decades [53], which included use in rice, wheat, maize, cassava, potato, and bean, among other crops, revealed that over 80% of the reported beneficial traits conferred by genes from wild relatives are involved in pest and disease resistance. Clearly, systematic efforts to bring genetic diversity from wild relatives into crop plants to incorporate a wider range of useful adaptations are required to increase the resiliency and productivity of crops.

Our data on common bean permit us to define introgression signals and differential haplotypes that, for the first time, can be combined to define domestication and putative adaptation loci. We confirmed a remarkable asymmetry of gene flow between wild and domesticated common bean subpopulations, as previously measured using microsatellite diversity [27]. Introgression signals between wild accessions might be disrupted as a consequence of the frequent hybridizations that have maintained high levels of genetic diversity (Additional file 1: Figure S13). In addition, introgression in domesticated genotypes from wild neighbors could be limited through selection against hybrids where wild traits, which are dominant or semi-dominant [75], are easily recognized by farmers. We cannot exclude, however, that the high introgression signals (f_d_ + d_XY_) between our defined subpopulations could actually underestimate the spans in each triad, which might cover larger portions of the genome if homogenous populations were tested.

As expected [28], domestication gene candidates do not overlap with introgressed regions since selective sweeps, i.e. homozygous regions that are rich in domestication-associated haplotypes, do not display signals of introgression. This is consistent with observations in other crops, such as maize, where domestication genes act as barrier loci for introgression events [29]. There is strikingly little overlap (65 PCGs) between our domestication PCG candidates and those reported previously [32]. This could be due to the absence of gene flow estimations in the previous model, as some of the reported loci that differentiate landraces from wild genotypes may not be the outcome of artificial selection, but rather represent admixture with other gene pools. Indeed, 40% of the published domestication gene candidates [32] recognized in the BAT93 gene set were within introgressed tracks in our test triads, implying that those might be neutral loci easily transferred between common bean subpopulations.

## Conclusions

First, the studies reported here demonstrate—based on genomic and metabolomics data, and reproductive isolation—how the ancestral nature of the AH wild populations in northern Peru and Ecuador derived from a dissemination and speciation event that preceded the *P. vulgaris* speciation event and the split of the latter species into two major geographic gene pools (Mesoamerican versus Andean), based on structural genomic, reproductive isolation, and metabolomic data. Second, they provide a genome-wide picture of the importance of gene flow in common bean, a predominantly autogamous species, in providing local adaptation both in wild and domesticated populations. They also confirm the predominance of the domesticated to wild gene flow. Third, they illustrate how the domesticated genome of common bean has been shaped not only by selection under domestication but also by gene flow from other common bean population and closely related species, like *P. dumosus* and *P. coccineus*. This gene flow may have led to adaptive genomic introgressions permitting the adaptation of cultivars to environments outside their centers of domestication. This capacity, as well as the high frequency of stress response genes, should be exploited to enrich the genetic diversity base of breeding programs.

## Methods

### Plant material

*Phaseolus vulgaris* cv. BAT93 is a breeding line developed at the International Center for Tropical Agriculture (CIAT, Cali, Colombia) and derived from a double cross involving four Mesoamerican genotypes: (Veranic × Tlalnepantla 64) × (Negro Jamapa × Tara). Its genome sequence was recently published [31] and was defined as our reference for downstream analyses. The biological material collected for this analysis included other important *P. vulgaris* accessions: eight wild Mesoamerican genotypes, selected according to their geographical distribution along the Mexican territory; one landrace from Chihuahua (Mexico); Jalo EEP558, a selection from the Andean landrace Jalo obtained by R. Guazelli at the Estação Experimental de Pato de Minas (Minas Gerais, Brazil); Faba Andecha, a Spanish cultivar of Andean origin selected based on its domesticated traits; an Andean wild accession from Argentina (G19901); and five accessions from Peru and Ecuador considered by other authors to represent the ancestral form of the species based on of their phaseolin isoform (PhI, [6]), all collected in the constrained location of the Amotape–Huancabamba Deflection [48]. Outside the *P. vulgaris* species, we selected 11 additional species covering most of the clade diversity of the genus, according to [11]. These species corresponded to the Tuerckheimii group (*P. hintonii* A. Delgado) and the unclassified group (*P. microcarpus* Mart.) from clade A and, from clade B, the groups Filiformis (*P. filiformis* Benth.), Lunatus (*P. lunatus* – lima bean), Polystachios (*P. polystachios* Britton and *P. maculatus* Scheele), Leptostachyus (*P. leptostachyus* Benth*.*), and Vulgaris (*P. coccineus*, *P. dumosus*, *P. costaricensis*, and *P. acutifolius*). Plants were grown under greenhouse conditions and young trifoliate leaves were collected for DNA extraction.

### DNA/RNA sequencing and mapping

DNA libraries were constructed and sequenced from both ends (paired-end reads) using the HiSeq (Illumina) technology at the Genomic Services Laboratory of LANGEBIO-CINVESTAV, Mexico. Reads of high quality (FastQC and FastxToolkit) were mapped with BWA v0.7.9a [76] using default parameters against the *P. vulgaris* BAT93 reference genome, as well as to a synteny-based pseudoassembly produced with SynMap at CoGe ([77]; https://genomevolution.org/coge/) of BAT93, taking the G19833 genome as scaffold with at least four contiguous syntenic CDSs between assembled tracks.

### Nuclear and chloroplast phylogenetic profiles

For each sequenced accession, individual–specific consensus sequences were generated and small variants (SNPs) were identified with ANGSD v0.614 [78] with the following options: major and minor alleles were inferred with doMaf = 1and doMajorMinor = 1; the genotype likelihood was calculated using the samtools method with GL = 1; positions at each chromosome covered in all 30 genotypes were controlled with minInd = 30; SNP-pval = 1e-6. Depth adjustments for SNP calling and consensus sequence reconstruction were done taking into account the sequencing depth of each accession: for all but four *P. vulgaris* accessions (Zacatecas, Oaxaca, Michoacán, Jalisco-Arandas) for which the depth threshold was set at five reads, a minimum of ten reads was required. Called SNPs in positions that were covered in all accessions were considered for further analyses.

From the collections of SNPs for each chromosome, singletons (unique SNPs for a particular genotype) were removed to avoid noisy signals derived from long-branch attraction effects (Additional file 2: Table S3). The filtered polymorphisms were then used to reconstruct phylogenetic trees based on the maximum likelihood (ML) approach. ML trees were reconstructed using the best-fitting evolutionary model, selected with PhyML v.3 [34] and using aLRT non-parametric SH branch support.

A 55-Kb chloroplast sequence was derived from scaffold00910 of the current BAT93 assembly, which was BLAST searched against the available genomic sequence of the plastid from *P. vulgaris* Negro Jamapa [79], displaying 99% identity. The consensus sequence of this scaffold was obtained as described above for the accessions belonging to the Vulgaris group and *P. hintonii*, as the outgroup. The 55-Kb plastid tracks were aligned and cleaned with TrimAl v1.3 [80]; the corresponding tree topology was constructed with the ML approach implemented in PhyML, using aLRT non-parametric SH branch support.

### Coalescent simulations

To obtain a temporal frame of the divergence between AH genotypes and the *P. vulgaris* clade, we conducted coalescent simulations using the same 55-Kb chloroplast sequence fragment as used in the phylogenetic analysis to avoid noisy signals from recombination events in the nuclear markers. We used the Bayesian approach implemented in BEAUti and BEAST v2.3.0 [81], considering only six genotypes: BAT93 and Jalo EEP558 (as representative genotypes of the MA and Andean genepools, respectively), one accession from Peru (G21245), *P. dumosus*, *P. costaricensis*, and *P. coccineus*. See Additional file 3 for more details on the priors for the simulations.

### Introgression signal

We combined two different parameters [48], the dynamic estimator of the degree of introgression between subpopulations (f_d_) and the absolute genetic distance (d_XY_). In principle, genomic regions that behave as f_d_ outliers can be distinguished as introgressed from ancestral variation if the absolute genetic distance d_XY_ is also reduced between a donor (P_3_) and a receptor population (P_2_), given that in the presence of gene flow, genomic windows coalesce more recently than the species split, so the magnitude of reduction in P_2_-P_3_ d_XY_ is greater than in the absence of recombination and hybridization. The f estimator was derived from the ABBA-BABA D statistic (Eq. 1a), and it assumes unidirectional gene flow from P_3_ to P_2_ (i.e. P_3_ is the donor and P_2_ is the recipient). In the case of the dynamic estimator f_d_, the denominator is calculated by defining a donor population (P_D_) for each site independently. For each site, P_D_ is the population (either P_2_ or P_3_) that has the higher frequency of the derived allele, thus maximizing the denominator and eliminating f estimates greater than 1 (Eq. 1b):

1 $$ \begin{array}{cccc}\hfill \mathsf{a}.\hfill & \hfill \hfill & \hfill \mathsf{b}.\hfill & \hfill \hfill \\ {}\hfill \hfill & \hfill \mathit{\mathsf{D}}\left({\mathit{\mathsf{P}}}_1,{\mathit{\mathsf{P}}}_2,{\mathit{\mathsf{P}}}_3, O\right)=\frac{{\displaystyle \sum }{C}_{\mathsf{ABBA}}\left(\mathit{\mathsf{i}}\right)\hbox{-} {C}_{\mathsf{BABA}}\left(\mathit{\mathsf{i}}\right)}{{\displaystyle \sum }{C}_{\mathsf{ABBA}}\left(\mathit{\mathsf{i}}\right)+{C}_{\mathsf{BABA}}\left(\mathit{\mathsf{i}}\right)}\begin{array}{ccc}\hfill \hfill & \hfill \hfill & \hfill \hfill \end{array}\hfill & \hfill \hfill & \hfill {\widehat{\mathit{\mathsf{f}}}}_{\mathit{\mathsf{d}}}=\frac{\mathsf{S}\left({\mathit{\mathsf{P}}}_1,{\mathit{\mathsf{P}}}_2,{\mathit{\mathsf{P}}}_3, O\right)}{\mathsf{S}\left({\mathit{\mathsf{P}}}_1,{\mathit{\mathsf{P}}}_{\mathsf{D}},{\mathit{\mathsf{P}}}_{\mathsf{D}}, O\right)}\hfill \end{array} $$

Eq. 1. Introgression estimators. (**a**) Patterson’s D statistic. C_ABBA_(i) and C_BABA_(i) are counts of either 1 or 0, depending on whether the pattern ABBA or BABA is observed at site I in the genomic block. P_1_/P_2_: receptor populations; P_3_: donor population; O: outgroup species. (**b**) Dynamic estimator. S: the difference between sums of ABBAs and BABAs, calculated using the frequency of the derived allele at each site in each population rather than binary counts; P_D_: the population (either P_2_ or P_3_) with the higher frequency of the derived allele that maximizes the denominator.

Introgressed blocks that belong to the top 5% f_d_ outliers that, at the same time, display d_XY_ values smaller than the mean d_XY_ across the whole genome were condensed using a custom R script to define genomic windows of at least three 5-Kb neighboring blocks. The parameters f_d_, d_XY_, π, and D were calculated for 5-Kb non-overlapping windows along the 11 linkage groups of the synteny-based pseudoassembly of BAT93, using the pipeline reported by [48] and available at http://datadryad.org/resource/doi:10.5061/dryad.j1rm6.

### Selection during domestication

Given that our sampling and sequencing strategies produced whole genomes from individuals belonging to different locations, we computed haplotype probabilities to identify haplotype clusters strongly associated with the domesticated phenotype. Since our sampling was biased to MA collections, particularly for wild *P. vulgaris* genotypes, we were not able to distinguish domestication haplotypes unique to the Andean cultivars, as we had one single wild accession from this area. However, we could differentiate haplotype clusters shared both by MA-domesticated and AN-domesticated cultivars, and those that emerged exclusively during the domestication process in MA.

For this purpose, we used the complete collection of SNPs of each *P. vulgaris* accession, including the genotypes from northern Peru and Ecuador as part of the wild subpopulation (19 genotypes in total) that were identified (Additional file 2: Table S3). The lists of non-unique SNPs from each chromosome were converted into tped files and then to bimbam format using Plink [82]. The resulting files were used as input for hapQTLv0.99 [51], a haplotype association method that relies on a hidden Markov model, and is suitable for large datasets to infer ancestral haplotypes and their loadings at each marker for each individual. With this algorithm, the local haplotype sharing (LHS)—the probability of two diploid individuals descending from the same ancestral haplotypes and thus a natural extension of identity by descent —can be quantified using the loadings. By testing whether the genetic similarity is associated with a particular phenotype, hapQTL is able to identify associations at each (core) marker between local haplotypes and phenotypes. For all hapQTL independent runs at each chromosome, we used two upper-layer clusters, two lower-layer clusters, and 20 steps in the EM runs using linear approximation; the rest of the parameters were kept as default. Two combinations of phenotypes were defined: (1) BAT93, Negro San Luis and Chihuahua labeled as “cases” of domestication in MA (DMA) and the other 16 genotypes (wild MA, AN and AH) as “controls;” and (2) BAT93, Negro San Luis, Chihuhahua, Jalo EEP558, and Faba Andecha labeled as “cases” of domestication in both COD, and the rest (wild MA, wild AN and AH) as “controls.” For each domestication phenotype, we permuted case–control labels once and computed Bayes factors, treating these as Bayes factors under the null. Based on the permutation tests, Bayes factors (bf1 and bf2) were filtered as follows: both COD, bf1 ≥ 3 and bf2 ≥ 3.5; DMA, bf1 ≥ 3.3 and bf2 ≥ 3.

Once selected based on their Bayes factors, SNPs were evaluated with SnpEff [83] to identify those markers located in the coding sequences (exons), regulatory regions (5’/3’ UTRs), or introns. We selected as domestication candidates those genes that contained at least two SNPs with high association factors to any domestication phenotype, and were affecting regulatory regions, had non-synonymous effects on the coding sequence, or altered splicing sites or stop codons.

### Gene Ontology enrichments

The functional description of genes falling within introgressed genomic windows and with domestication haplotypes was analyzed [31]. Gene Ontology enrichments for each case were performed using the topGO package implemented in Bioconductor [84], using the classic Fisher’s exact test with a maximum *p* value of 0.05.

### Sample preparation and extraction for metabolomic profiling

Young trifoliate leaves from *P. vulgaris*, *P. pseudovulgaris*, and *P. coccineus* were collected and immediately frozen in liquid nitrogen. The leaves were then lyophilized and finely ground (<300 μm) using a Mixer Mill MM 400 (Retsch®). Subsequently, extracts were prepared mixing 50 mg plant powder in 1000 μL methanol and formic acid solution (75% *v/v* and 0.15% *v/v*, respectively). The mixture was sonicated for 15 min in a water bath at maximum frequency and centrifuged at 10,000 *g* for 10 min at 4 °C. The supernatant was filtered through a 0.22-μm filter before analysis by DIESI-MS. All samples were prepared by triplicate and analyzed immediately.

### Mass spectrometry

For DIESI-MS analysis, the methanolic extracts of *Phaseolus* leaves were injected directly (flow rate 10 μL/min^–1^) to a mass spectrometer equipped with an electrospray ionization source and a single quadrupole analyser (Micromass ZQ, Waters Corps. Mexico). Mass spectra were acquired in positive mode with the following settings: capillary voltage 2.75 kV, cone voltage 35 V, and extractor voltage 4 V. The desolvation gas was set to 400 L/h^–1^ at a temperature of 250 °C. The cone gas was set to 50 L/h^–1^, and the source temperature to 120 °C. Continuum data were acquired in a range of 50–1300 *m*/*z* during 1 min with a scan time of 10 s and an inter-scan time of 0.1 s.

### Non-targeted metabolite profiling

For non-targeted metabolite profiling, samples previously extracted with methanol were reconstituted in a mixture of methanol/de-ionized water/formic acid (75:24.85:0.15 [*v*/*v/v*]) and filtered through a 0.2-μm filter. Chromatographic separation was achieved on an Acquity UPLC System (Waters, Milford) using a BEH C18 2.1 × 50 mm, 1.7-um column maintained at 40 °C. Samples were injected (10 μL) and elution of compounds was performed at a flow rate of 0.5 mL/min as follows: mobile phase A: de-ionized water containing 0.1% formic acid; mobile phase B: acetonitrile containing 0.15% formic acid. The gradient program was isocratic for the first 30 s, then a linear gradient increase to 30% of solvent B at 2 min, 40% of B at 4 min, 40% of B at 6 min, and 70% of B at 10 min, with 1 min for column washing and 4 min for column re-equilibration. The mass spectrometer comprised an orthogonal QTOF Synapt G1 (Waters, UK) operated under the following conditions: electrospray ionization in positive mode, capillary voltage at 3.0 kV, cone voltage 46 V, extractor voltage 4.0 V, source and desolvation temperature were 120 and 300 °C, respectively. Cone and desolvation gas flow was nitrogen at a flow rate of 20 L/h and 800 L/min. Leucine-enkephaline (M + H) + = 556.2771 was infused at a flow rate of 5 μL/min at concentration of 2 ng/mL during acquisition as internal mass calibrant to correct for mass drift.

### Metabolomic data analysis

Prior to data analysis, the.*raw* native data format for each spectrum were transformed to standard mass spectrometry.*mzML* format employing msconvert [85]. The spectrum data were then processed using a workflow designed in R (http://www.rproject.org) with the package MALDIquant [86] as follows:*.mzML* data import, summarizing all scans of each sample, smoothing by a Savitzky–Golay filter, and peak alignment/detection for comparison of peaks across different spectra. In total, 318 high quality intensity values of ions were used for statistical analysis. We employed a hierarchical clustering analysis (HCA) approach for the generation of metabolic heatmaps to evaluate the differences in the fingerprinting data. To find the most important ions, we generated a Random Forest Tree model for classification in the R package “Rattle” [87]. LC-MS/MS data were analyzed using MS-672DIAL software v2.06 [88]. Peak annotation was performed comparing fragment mass spectra with MassBank, ReSpect ESI, and MS/MS libraries in positive ion mode.

## Additional files

Additional file 1: Supplementary figures **S1–S4** and **S11–S21**. (PDF 5628 kb)

Additional file 2: **Tables S1–S3.** **Table S12** and **Table S20**. (PDF 175 kb)

Additional file 3: Supplementary text, **Tables S4–S11** and **Figures S5–S10.** Coalescent simulations. (PDF 693 kb)

Additional file 4: **Table S13.** Domestication gene candidates common to 2CODs. (XLSX 92 kb)

Additional file 5: **Table S14.** Domestication gene candidates specific to Mesoamerican varieties. (XLSX 100 kb)

Additional file 6: **Table S15.** PCGs and functional categories introgressing between wild subpopulations. (XLSX 109 kb)

Additional file 7: **Table S16.** PCGs and functional categories introgressing from wild into domesticated subpopulations. (XLSX 99 kb)

Additional file 8: **Table S17.** PCGs and functional categories introgressing from domesticated into wild subpopulations. (XLSX 149 kb)

Additional file 9: **Table S18.** GO terms associated to domestication PCGs common to 2CODs. (XLSX 52 kb)

Additional file 10: **Table S19.** GO terms associated to domestication PCGs specific to Mesoamerican varieties. (XLSX 56 kb)
