# Supplementary material for: Genomic history of the origin and domestication of common bean unveils its closest sister species
Source: Genome Biol. 2017 Mar 29;18:60. doi: 10.1186/s13059-017-1190-6 (PMC5370463; doi:10.1186/s13059-017-1190-6)
Supplement: Supplementary file 1 — Supplementary figures S1–S4 and S11–S21. (PDF 5628 kb) [file 13059_2017_1190_MOESM1_ESM.pdf]

Genomic history of the origin and domestication of common bean unveils its closest sister species

Additional file 1.

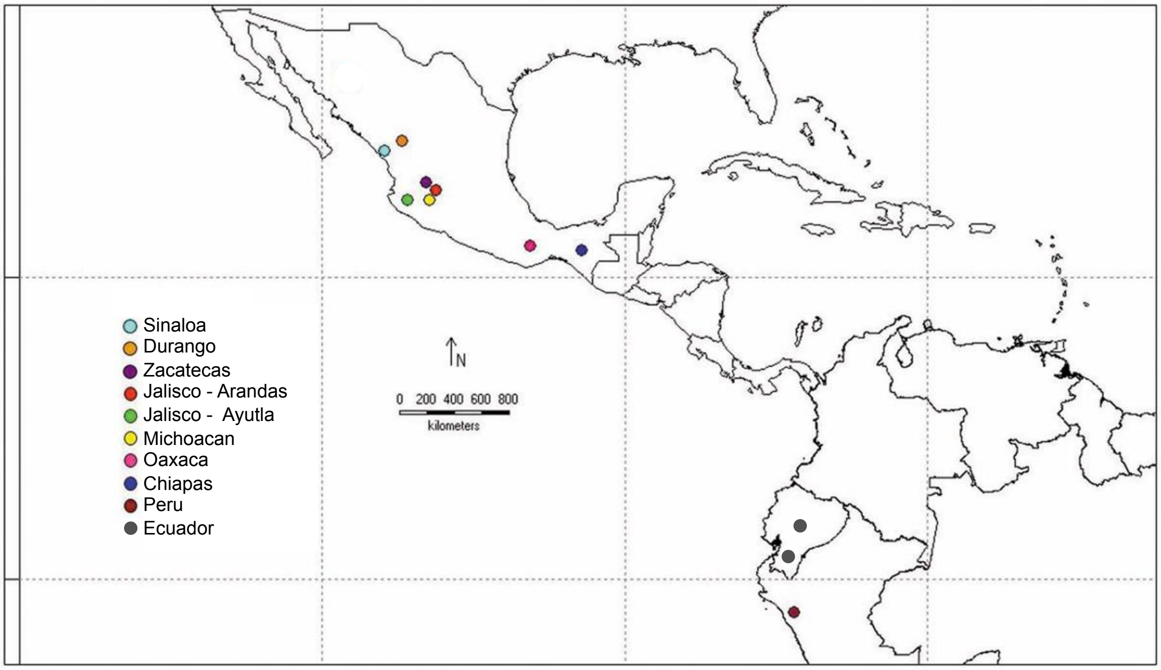

Figure S1. Geographic origin of wild *P. vulgaris* accessions.

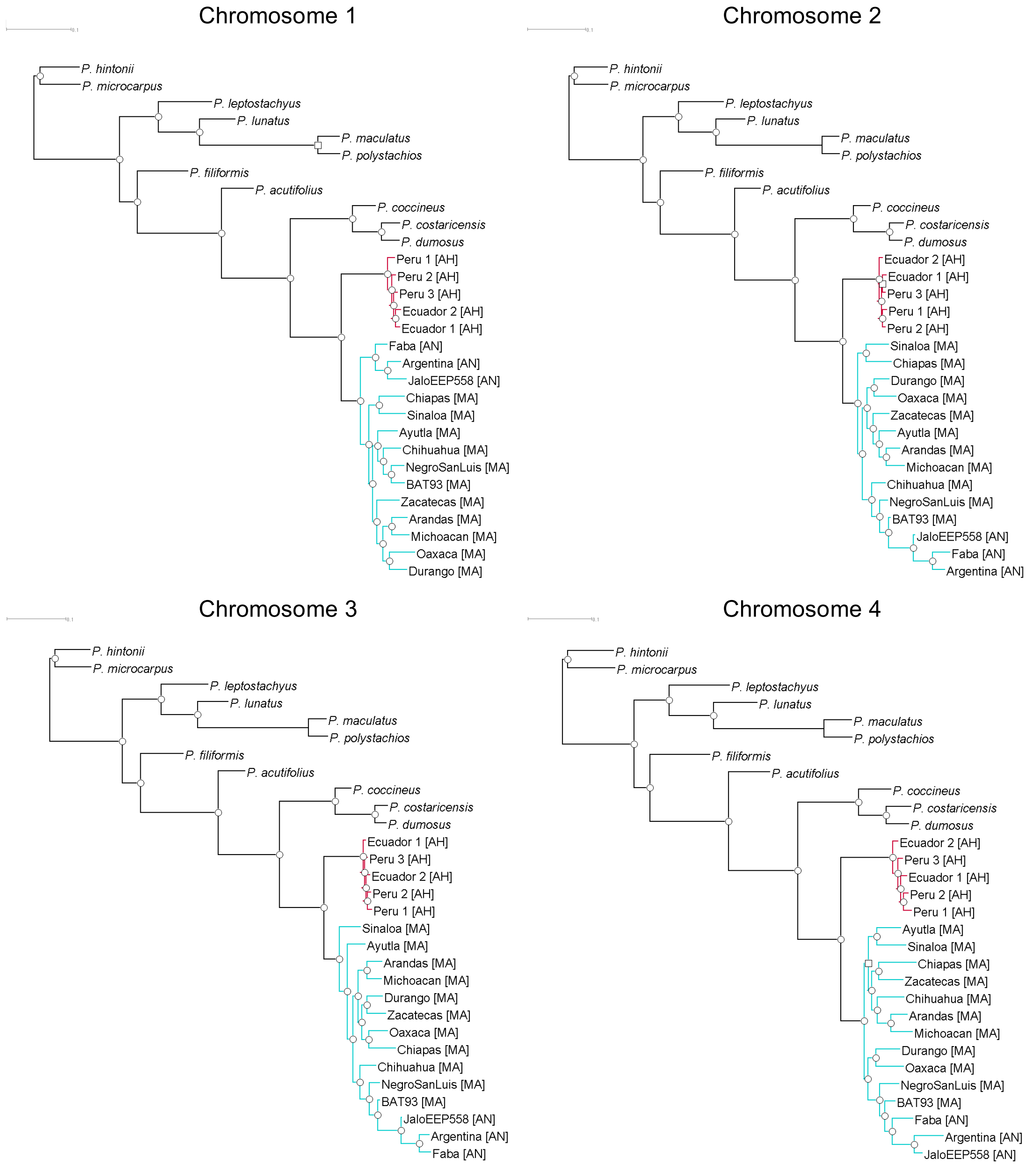

Figure S2. Phylogenetic trees produced using the ML method and SH-aLRT branch support implemented in PhyML, considering non-unique SNPs from chromosomes 1 to 4 according to the pseudoassembly of *P. vulgaris* cv. BAT93 against *P. vulgaris* G19833. Branch support: aLRT>0.95, circles; aLRT [0.75-0.95], squares.

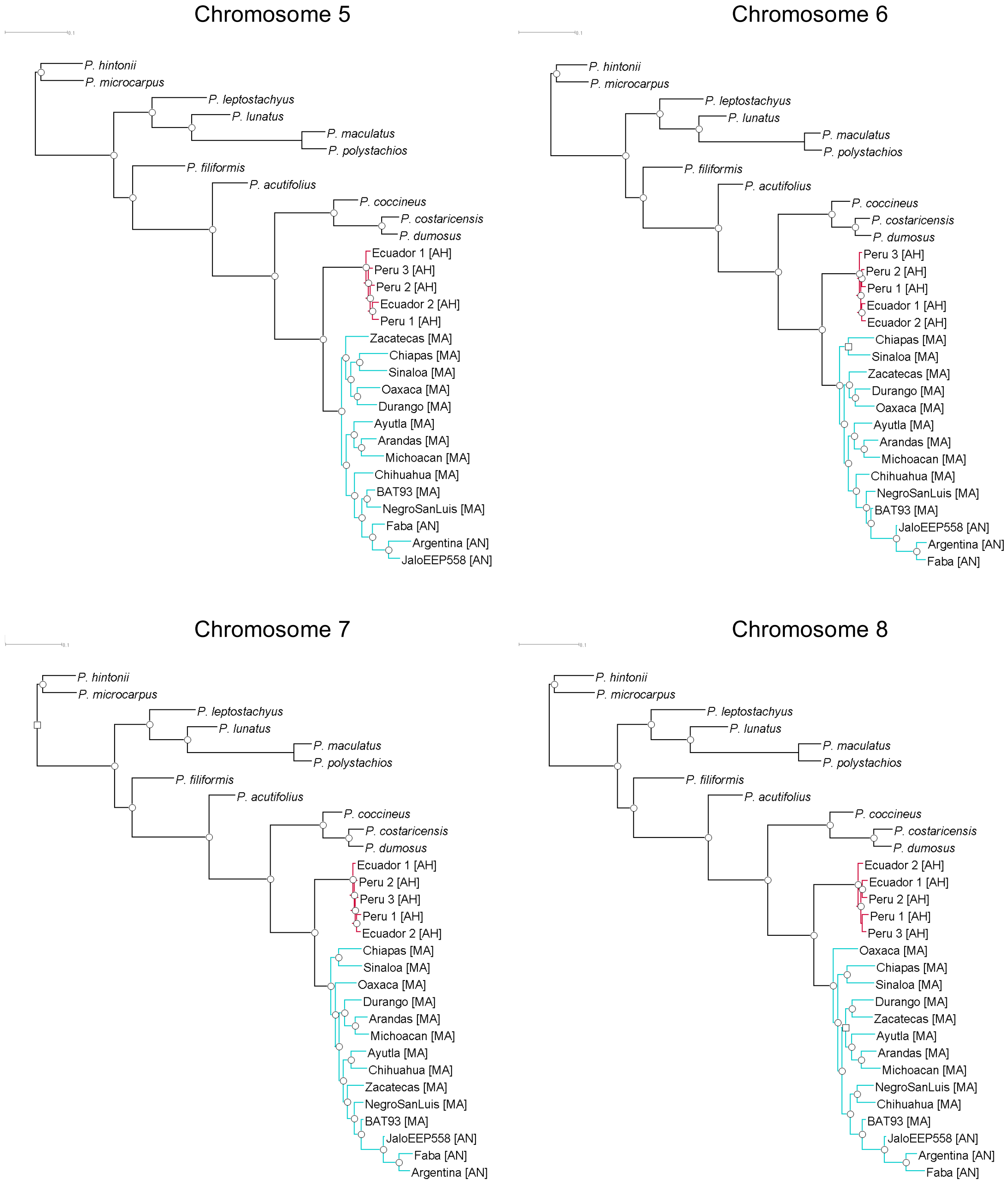

Figure S3. Phylogenetic trees produced using the ML method and SH-aLRT branch support implemented in PhyML, considering non-unique SNPs from chromosomes 5 to 8 according to the pseudoassembly of *P. vulgaris* cv. BAT93 against *P. vulgaris* G19833. Branch support: aLRT>0.95, circles; aLRT [0.75-0.95], squares.

Chromosome 9

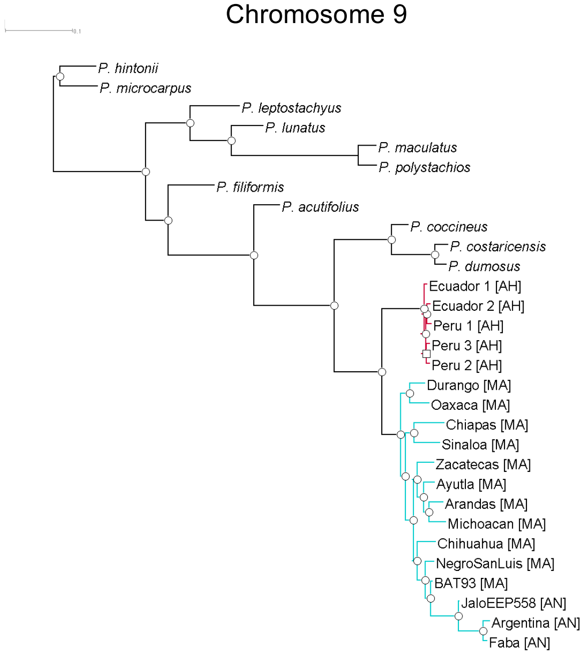

Chromosome 10

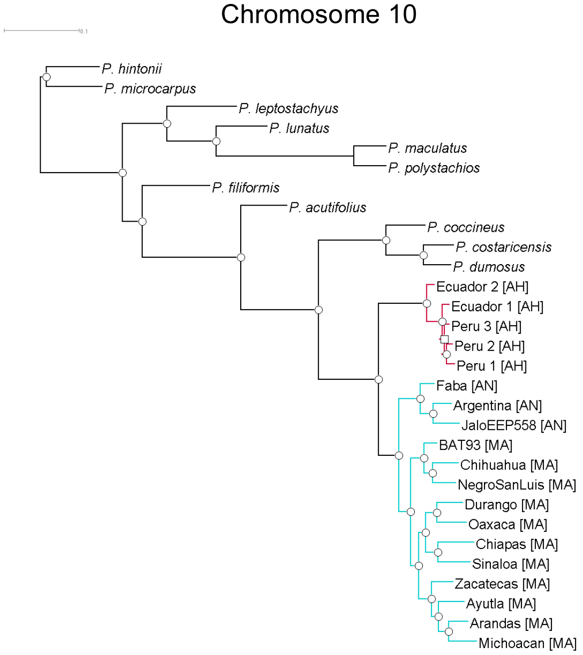

Chromosome 11

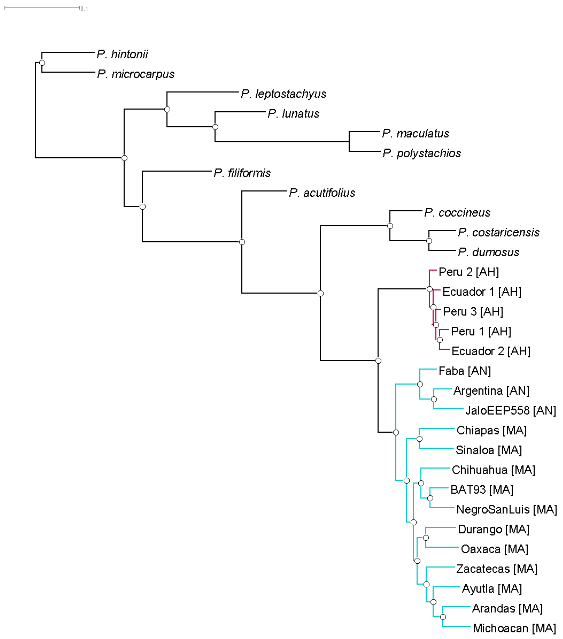

Figure S4. Phylogenetic trees produced using the ML method and SH-aLRT branch support implemented in PhyML, considering non-unique SNPs from chromosomes 9 to 11 according to the pseudoassembly of *P. vulgaris* cv. BAT93 against *P. vulgaris* G19833. Branch support: aLRT>0.95, circles; aLRT [0.75-0.95], squares.

a.

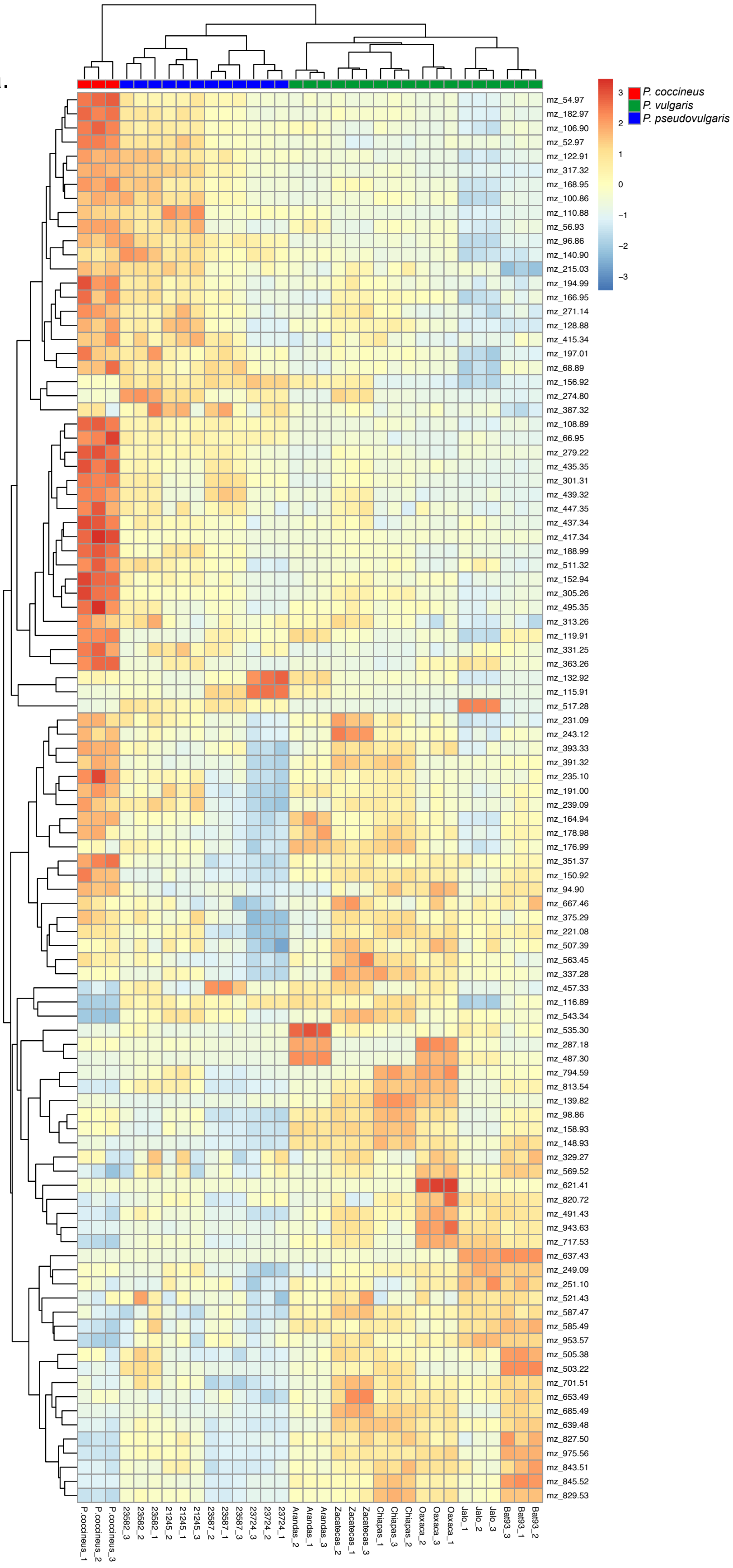
P. coccineus
P. vulgaris
P. pseudovulgaris
3
2
1
0
−1
−2
−3
mz_54.97
mz_182.97
mz_106.90
mz_52.97
mz_122.91
mz_317.32
mz_168.95
mz_100.86
mz_110.88
mz_56.93
mz_96.86
mz_140.90
mz_215.03
mz_194.99
mz_166.95
mz_271.14
mz_128.88
mz_415.34
mz_197.01
mz_68.89
mz_156.92
mz_274.80
mz_387.32
mz_108.89
mz_66.95
mz_279.22
mz_435.35
mz_301.31
mz_439.32
mz_447.35
mz_437.34
mz_417.34
mz_188.99
mz_511.32
mz_152.94
mz_305.26
mz_495.35
mz_313.26
mz_119.91
mz_331.25
mz_363.26
mz_132.92
mz_115.91
mz_517.28
mz_231.09
mz_243.12
mz_393.33
mz_391.32
mz_235.10
mz_191.00
mz_239.09
mz_164.94
mz_178.98
mz_176.99
mz_351.37
mz_150.92
mz_94.90
mz_667.46
mz_375.29
mz_221.08
mz_507.39
mz_563.45
mz_337.28
mz_457.33
mz_116.89
mz_543.34
mz_535.30
mz_287.18
mz_487.30
mz_794.59
mz_813.54
mz_139.82
mz_98.86
mz_158.93
mz_148.93
mz_329.27
mz_569.52
mz_621.41
mz_820.72
mz_491.43
mz_943.63
mz_717.53
mz_637.43
mz_249.09
mz_251.10
mz_521.43
mz_587.47
mz_585.49
mz_953.57
mz_505.38
mz_503.22
mz_701.51
mz_653.49
mz_685.49
mz_639.48
mz_827.50
mz_975.56
mz_843.51
mz_845.52
mz_829.53
P.coccineus_1
P.coccineus_2
P.coccineus_3
23582_3
23582_2
23582_1
21245_2
21245_1
21245_3
23587_2
23587_1
23587_3
23724_3
23724_2
23724_1
Arandas_2
Arandas_1
Arandas_3
Zacatecas_2
Zacatecas_1
Zacatecas_3
Chiapas_1
Chiapas_3
Chiapas_2
Oaxaca_2
Oaxaca_3
Oaxaca_1
Jalo_1
Jalo_2
Jalo_3
Ba93_3
Ba93_1
Ba93_2

b.

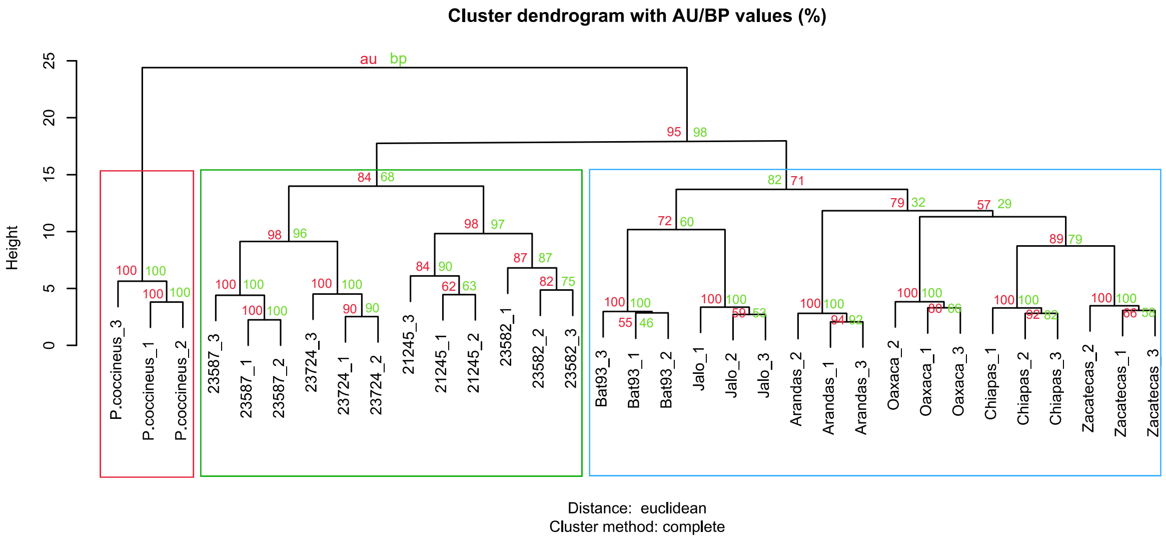

Figure S11. Metabolic profile of *Phaseolus* accessions considering the top 100 most abundant metabolites. a. Hierarchical clustering/bootstrap tree of *Phaseolus* accessions; AU (Approximately Unbiased) and bootstrap probabilities are highlighted in red and green, respectively. Coloured boxed enclose the independent clades of *P. coccineus* (red), AH - *P. pseudovulgaris* accessions (green) and *P. vulgaris* (blue). b. Metabolic heatmap and clustering of the accessions.

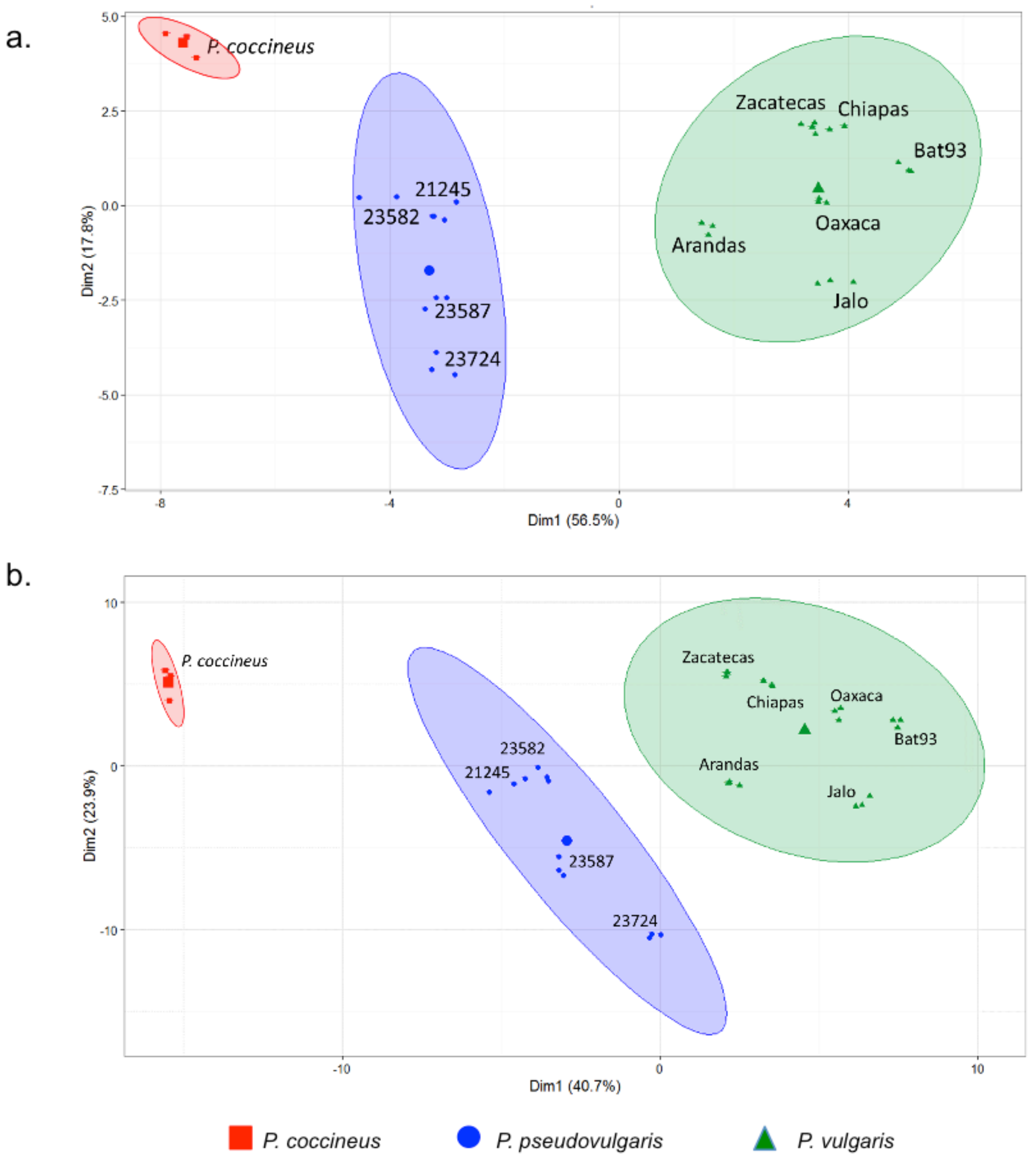

Figure S12. Principal component analysis of *Phaseolus* accessions considered in the metabolomics fingerprinting. a. PCA using the 30 most informative metabolites obtained following a machine learning approach. b. PCA using the 100 most abundant metabolites in the screening.

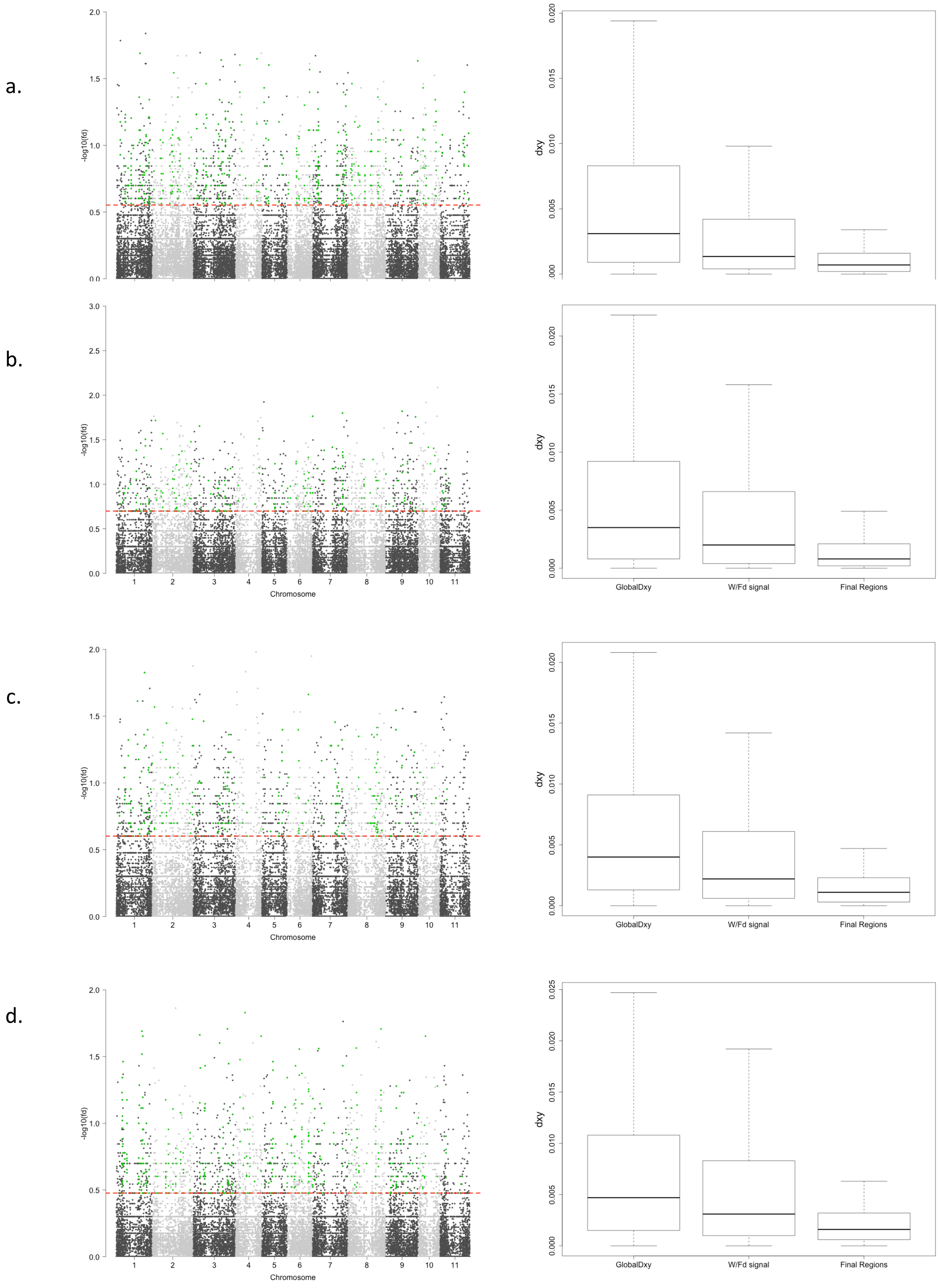

Figure S13. Introgression signal between wild *P. vulgaris* subpopulations. f_d values in 5kb non-overlapping windows of each chromosome are represented in Manhattan plots on left panels; the red threshold lines show the top 5% f_d outliers in each comparison, and strong signals of introgression (f_d+d_{XY}) are highlighted in green. The directionality of genomic flux corresponds to: North towards West (a), South towards North (b), South towards West (c), North towards Center (d). Absolute genetic divergence (d_{XY}) calculated at the genome-wide scale (left), in f_d 5% outliers (middle) and regions with introgression signal (f_d+d_{XY}, right) are shown in boxplots on the right panel of each comparison.

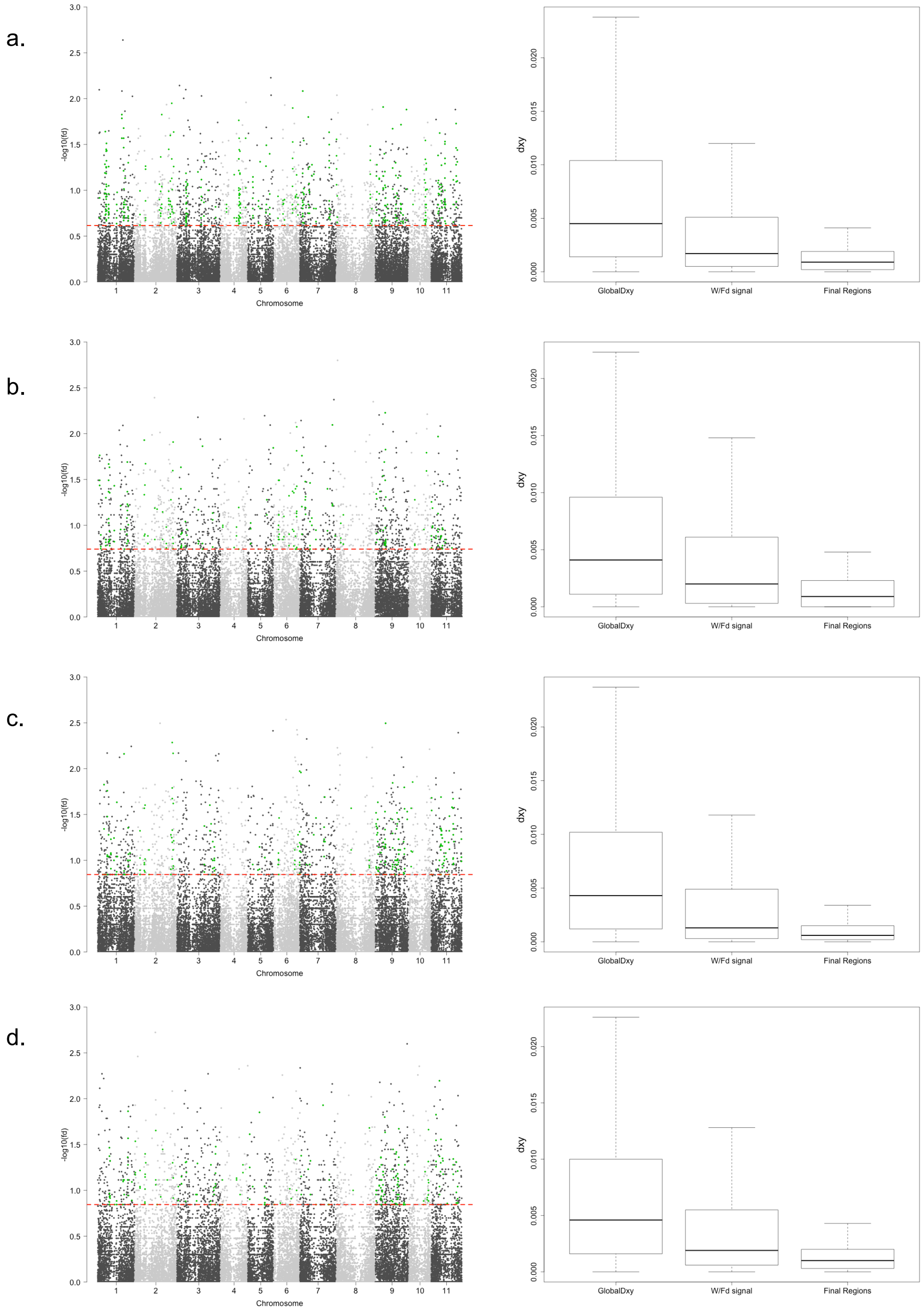

Figure S14. Introgression signal between wild and domesticated (DMA) *P. vulgaris* subpopulations. f_d values in 5kb non-overlapping windows of each chromosome are represented in Manhattan plots on left panels; the red threshold lines show the top 5% f_d outliers in each comparison, and strong signals of introgression (f_d $+d_{XY}$) are highlighted in green. The directionality of genomic flux corresponds to: North towards DMA (a), South towards DMA (b), Center towards DMA (c), West towards DMA (d). Absolute genetic divergence (d_{XY}) calculated at the genome-wide scale (left), in f_d 5% outliers (middle) and regions with introgression signal (f_d $+d_{XY}$, right) are shown in boxplots on the right panel of each comparison.

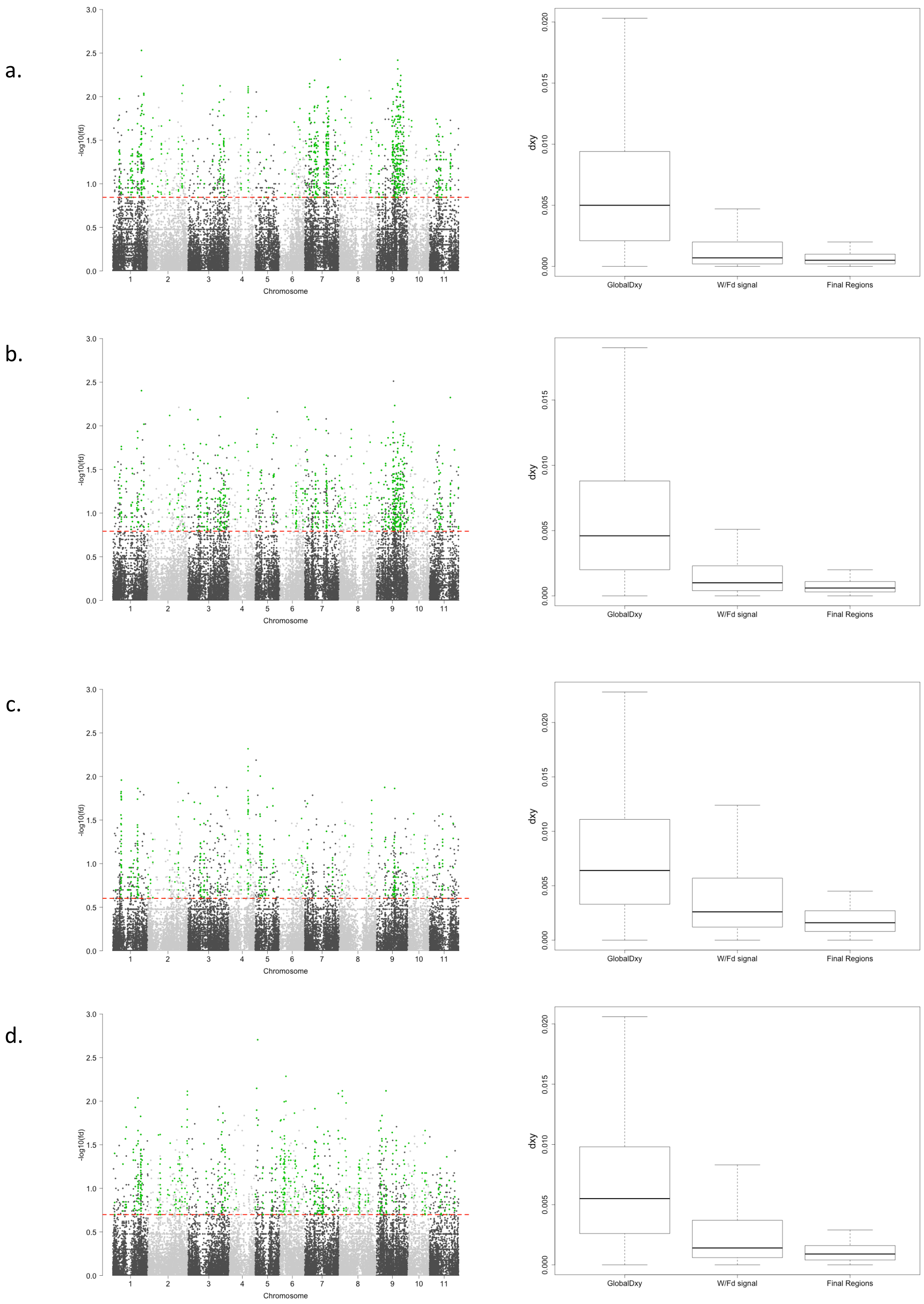

Figure S15. Introgression signal between domesticated (DMA) and wild *P. vulgaris* subpopulations. f_d values in 5kb non-overlapping windows of each chromosome are represented in Manhattan plots on left panels; the red threshold lines show the top 5% f_d outliers in each comparison, and strong signals of introgression (f_d +d_{XY}) are highlighted in green. The directionality of genomic flux corresponds to: DMA towards Center (a), DMA towards West (b), DMA towards North (c), DMA towards South (d). Absolute genetic divergence (d_{XY}) calculated at the genome-wide scale (left), in f_d 5% outliers (middle) and regions with introgression signal (f_d +d_{XY}, right) are shown in boxplots on the right panel of each comparison.

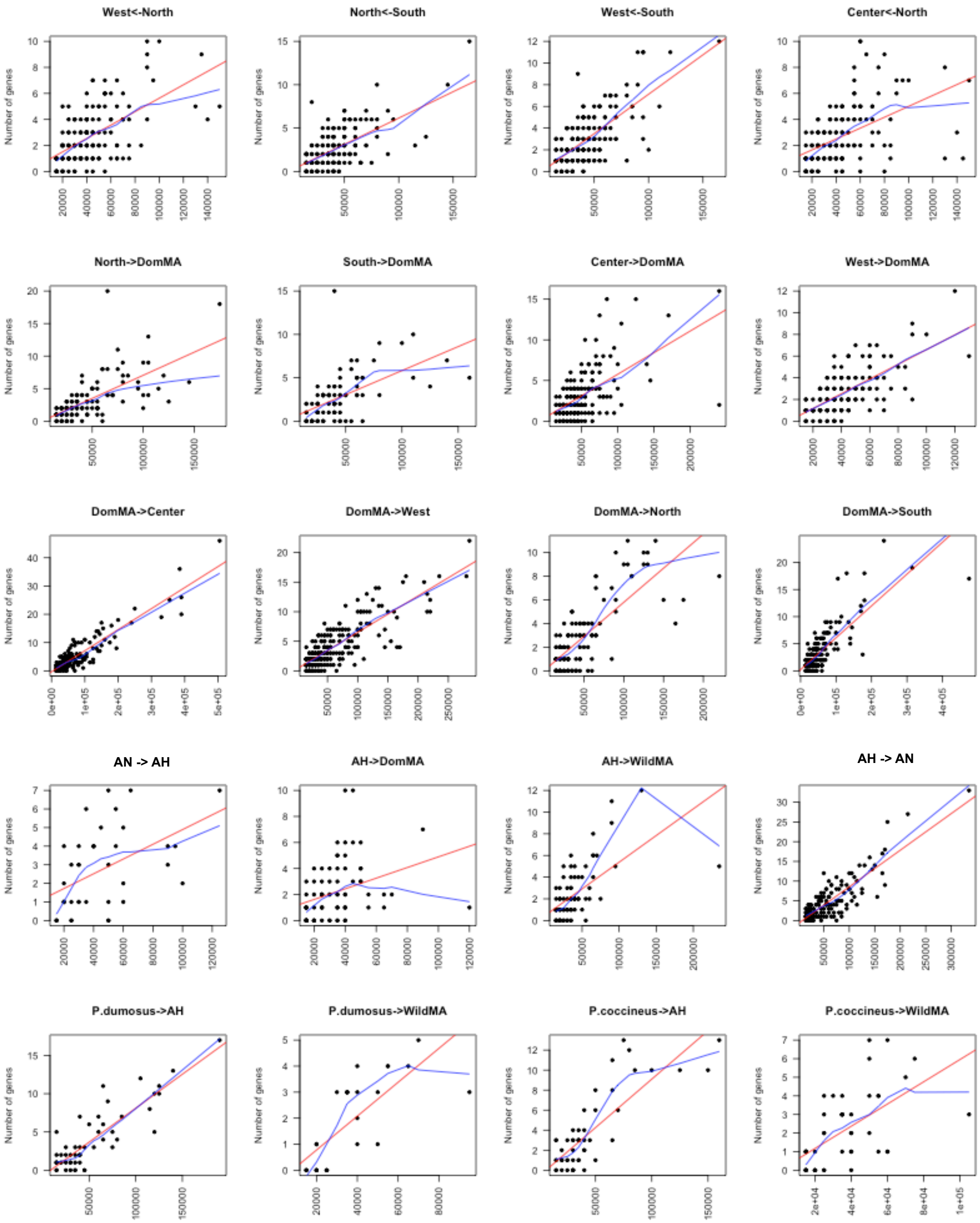

Figure S16. Introgressed PCG. The number of genes encoded in each introgressed block is represented in scatter plots. Coloured lines: linear (red) and local (blue) regressions.

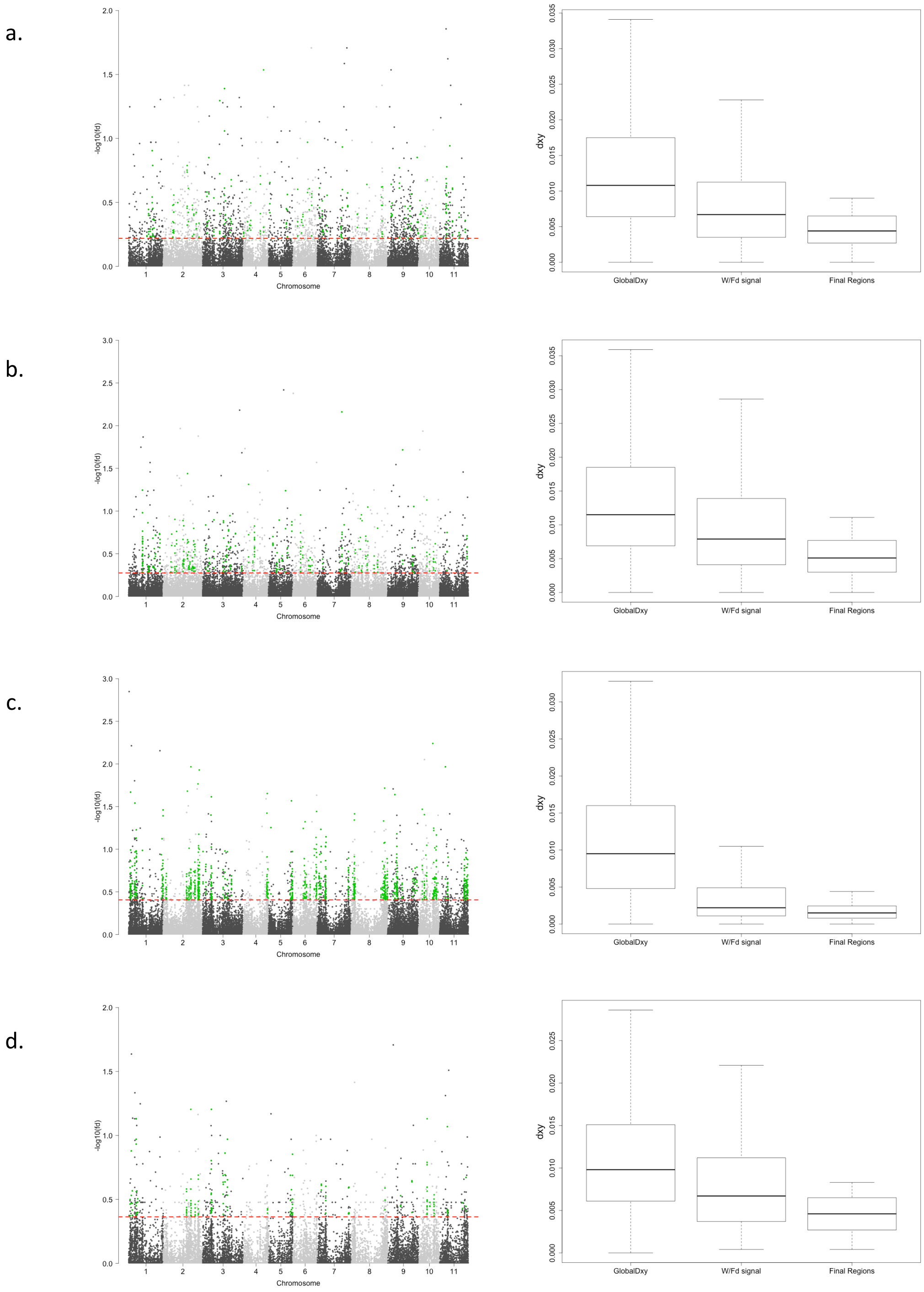

Figure S17. Introgression signal between *P. pseudovulgaris* and *P. vulgaris* subpopulations. f_d values in 5kb non-overlapping windows of each chromosome are represented in Manhattan plots on left panels; the red threshold lines show the top 5% f_d outliers in each comparison, and strong signals of introgression (fd+d_{XY}) are highlighted in green. The directionality of genomic flux corresponds to: AH towards DMA (a), AH towards WMA (b), AH towards AND (c), AN towards AH (d). Absolute genetic divergence (d_{XY}) calculated at the genome-wide scale (left), in f_d 5% outliers (middle) and regions with introgression signal (f_d +d_{XY}, right) are shown in boxplots on the right panel of each comparison.

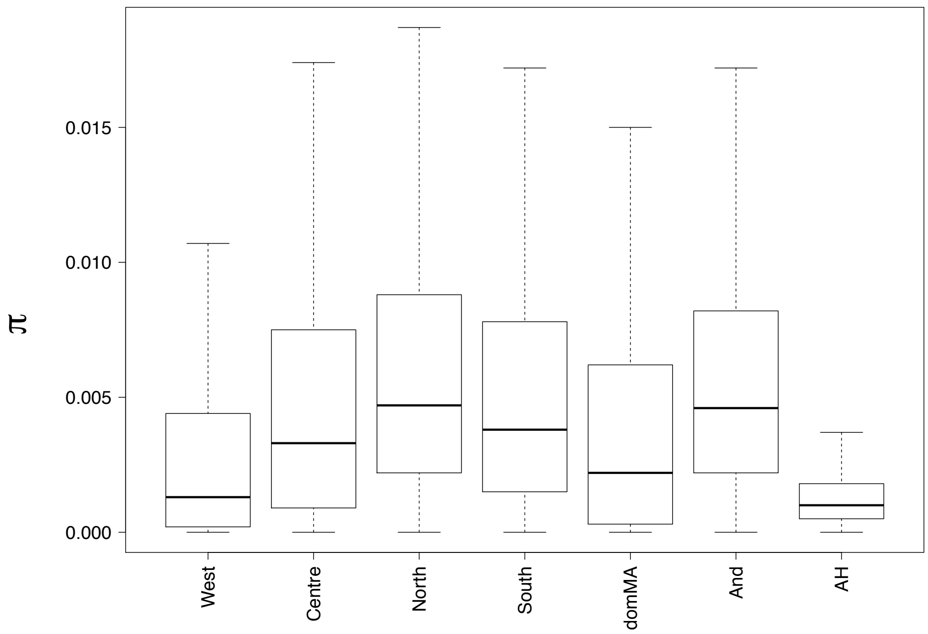

Figure S18. Genome-wide nucleotide diversity (π) across *P. vulgaris* subpopulations.

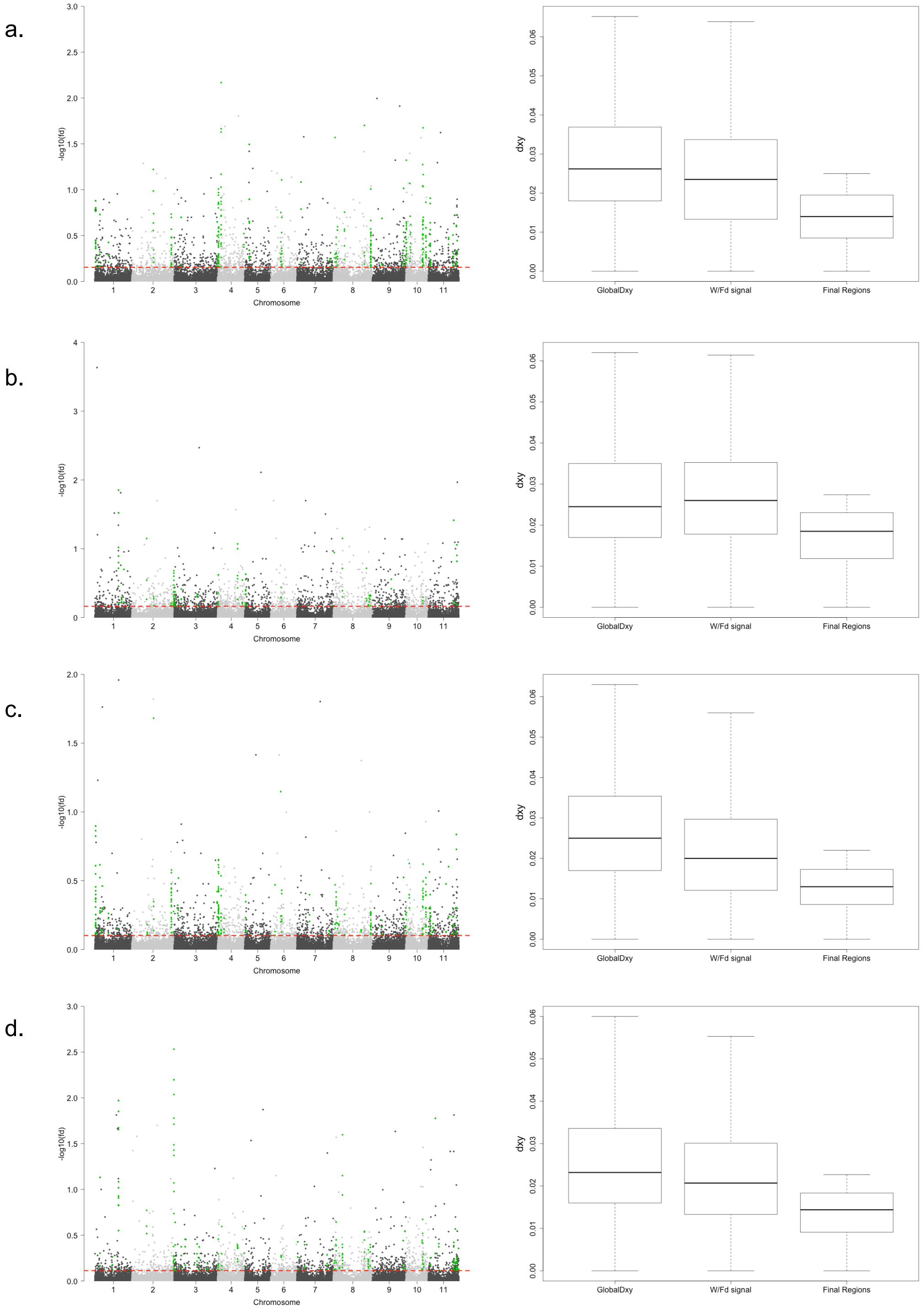

Figure S19. Introgression signal between *Phaseolus* spp. and *P. vulgaris* / *P. pseudovulgaris* subpopulations. f_d values in 5kb non-overlapping windows of each chromosome are represented in Manhattan plots on left panels; the red threshold lines show the top 5% f^d outliers in each comparison, and strong signals of introgression (f_d +d_{XY}) are highlighted in green. The directionality of genomic flux corresponds to: *P. dumosus/P. costaricensis* towards AH (a), *P. dumosus/P. costaricensis* towards WMA (b), *P. coccineus* towards AH (c), *P. coccineus* towards WMA (d). Absolute genetic divergence (d_{XY}) calculated at the genome-wide scale (left), in f_d 5% outliers (middle) and regions with introgression signal (f_d +d_{XY}, right) are shown in boxplots on the right panel of each comparison.

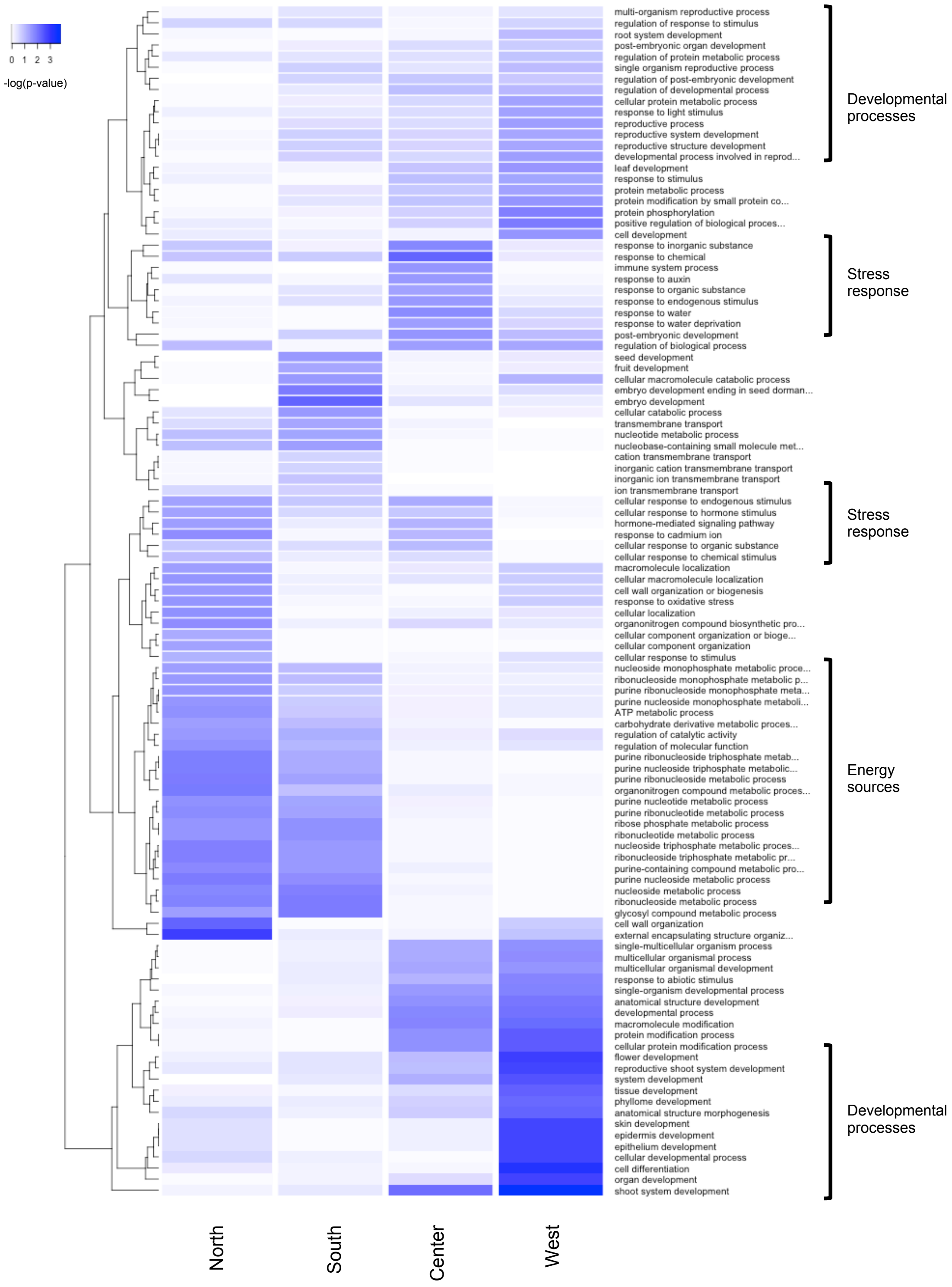

Figure S20. Enriched categories among PCGs introgressed from wild MA subpopulations into domesticated genotypes.

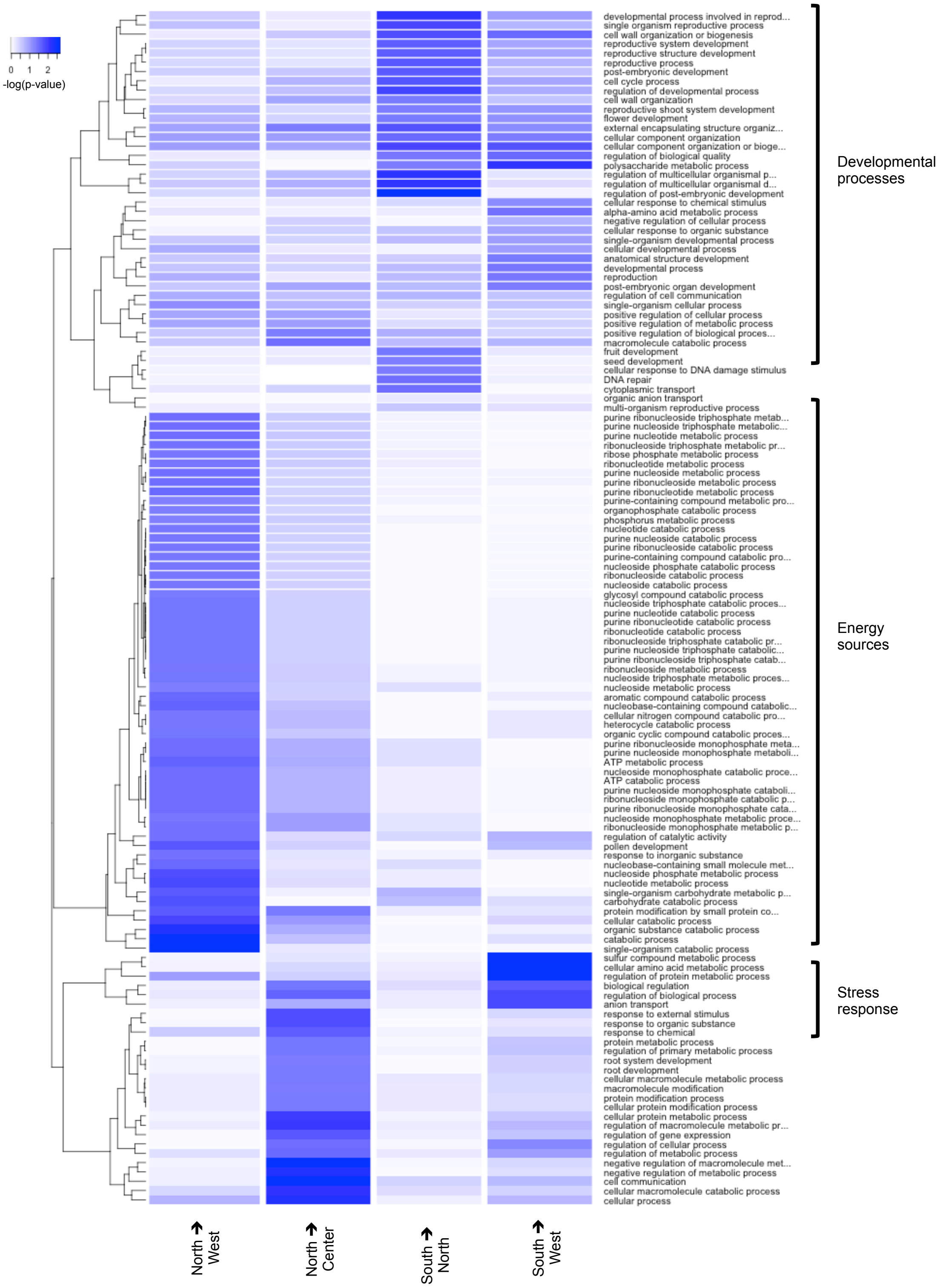

Figure S21. Enriched categories among PCGs introgressed between wild MA subpopulations.
