# Supplementary material for: Genomic history of the origin and domestication of common bean unveils its closest sister species
Source: Genome Biol. 2017 Mar 29;18:60. doi: 10.1186/s13059-017-1190-6 (PMC5370463; doi:10.1186/s13059-017-1190-6)
Supplement: Supplementary file 2 — Tables S1–S3. Table S12 and Table S20. (PDF 175 kb) [file 13059_2017_1190_MOESM2_ESM.pdf]

Genomic history of the origin and domestication of common bean unveils its closest sister species

Additional file 2.

Table S1. Plant material.

Species	Clade	Group	ID	Country	State/ Province	Gene pool	Altitude (m)	100 seed weight (g)
P. vulgaris	B	Vulgaris	G24392	Mexico	Jalisco/ Arandas	MA	2020	5.0
P. vulgaris	B	Vulgaris	G24377	Mexico	Michoacan/ Cojumatlan	MA	1700	4.0
P. vulgaris	B	Vulgaris	G50368	Mexico	Oaxaca/ Tlacolula De Matamoros	MA	1480	6.8
P. vulgaris	B	Vulgaris	G23551	Mexico	Sinaloa/ Concordia	MA	710	4.2
P. vulgaris	B	Vulgaris	G12967	Mexico	Jalisco/ Ayutla	MA	-	5.0
P. vulgaris	B	Vulgaris	G23463	Mexico	Chihuahua/ Yepachic	MA	1530	9.4
P. vulgaris	B	Vulgaris	G23550	Mexico	Zacatecas/ Moyahua De E.	MA	1700	6.3
P. vulgaris	B	Vulgaris	G24594	Mexico	Chiapas/ Ixtapa	MA	1400	5.4
P. vulgaris	B	Vulgaris	G23556	Mexico	Durango/Durango	MA	1860	8.5
P. vulgaris	B	Vulgaris	G23939 Negro San Luis	Mexico	-	MA		> 20.0
P. vulgaris	B	Vulgaris	Faba Andecha	Spain	-	AND		100.0
P. vulgaris	B	Vulgaris	Jalo EEP558 G09603	Brazil	Minas Gerais/ Patos De Minas	AND	-	41.0
P. vulgaris	B	Vulgaris	G19901	Argentina	Tucuman/ El Mollar	AND	1900	9.0
P. vulgaris	B	Vulgaris	G21244	Peru (1)	Cajamarca/ San Pablo	AH	2020	9.0
P. vulgaris	B	Vulgaris	G21245	Peru (2)	Cajamarca/ San Miguel	AH	1790	10.5
P. vulgaris	B	Vulgaris	G23587	Peru (3)	Cajamarca/ Chota	AH	1250	9.0
P. vulgaris	B	Vulgaris	G23724	Ecuador (1)	Loja/ Macará	AH	960	11.0
P. vulgaris	B	Vulgaris	G23582	Ecuador (2)	Chimborazo/ Alausi	AH	1710	8.4
P. costaricensis	B	Vulgaris	G40811A	Costa Rica	Cartago/ Cartago		1510	18.8
P. dumosus	B	Vulgaris	G36043	Mexico	Chiapas/ Ixtapa		1680	70.3
P coccineus	B	Vulgaris		Mexico	Querétaro/ La Joya		-	-
P. maculatus	B	Polystachios	PL-8841	Mexico	-	-	-	-
P. polystachios	B	Polystachios	G40782	Mexico	-	-		8.4
P. leptostachyus	B	Leptostachyus	PL-8829	Mexico	-	-	-	-
P. filiformis	B	Filiformis	-	Mexico	Baja California Sur	-	-	-
P. lunatus	B	Lunatus	PL-8834	Mexico	-	-	-	-
P. acutifolius	B	Vulgaris	-	Mexico	Chiapas	-	-	-
P. hintonii	A	Tuerckheimii	-	Mexico	Edo. Mexico	-	-	-
P. microcarpus	A	-	PL-8844	Mexico	-	-	-	-

Table S2. Genome coverage.

Group	Species	Reads (e^6)	Theoretical Coverage[a]	Depth of Coverage[b]	Breath of Coverage[c]
Vulgaris	*P. dumosus*	359	55.2x	27.5x	85%
	P. costarricencis	317	48.8x	29.4x	87%
	P. acutifolius	242	37.2x	19.6x	60%
	P. coccineus	285	43.8x	31.5x	88%
	P. vulgaris (G24392)	90	13.8x	6.4x	91%
	P. vulgaris (G24377)	142	21.8x	9.4x	94%
	P. vulgaris (G50368)	91	14.0x	7.5x	92%
	P. vulgaris (G23556)	170	26.2x	22.4x	94%
	P. vulgaris (G23939)	129	19.8x	18.1x	96%
	P. vulgaris (G23551)	173	26.6x	20.2x	94%
	P. vulgaris (G12967)	155	23.8x	18.3x	94%
	P. vulgaris (G23463)	198	30.5x	17.9x	95%
	P. vulgaris (G23550)	121	18.6x	12.4x	90%
	P. vulgaris (G24594)	342	52.6x	25.0x	95%
	P. vulgaris (Faba Andecha)	226	34.8x	28.6x	93%
	P. vulgaris (G19901)	127	19.5x	14.1x	93%
	P. vulgaris (G09603)	274	42.2x	36.6x	98%
	P. vulgaris (G21244)	338	52.0x	48.6x	92%
	P. vulgaris (G21245)	249	38.3x	34.3x	92%
	P. vulgaris (G23587)	299	46.1x	37.4x	93%
	P. vulgaris (G23724)	236	36.3x	15.7x	67%
	P. vulgaris (G23582)	189	29.1x	17.9x	92%
**Leptostachyus**	*P. leptostachyus*	258	39.7x	22.0x	77%
**Lunatus**	*P. lunatus*	200	30.8x	14.8x	68%
**Polystachios**	*P. polystachios*	293	45.1x	19.7x	67%
	P. maculatus	192	29.6x	15.4x	66%
**Filiformis**	*P. filiformis*	131	20.1x	15.7x	67%
**Tuerckhemii**	*P. hintonii*	182	28.0x	11.8x	59%
**ND**	*P. microcarpus*	190	29.3x	12.8x	56%

[a] Calculated over 650 Mb of theoretical genome length.
[b] Calculated over 556.4 Mb of BAT93 genome assembly.
[c] Calculated over the evelen synteny-based pseudo-assembled linkage groups after 'N' removal.

Table S3. SNPs detected in each chromosome.

	Chr1	Chr2	Chr3	Chr4	Chr5	Chr6
**Non-unique**	705048	838484	823694	408724	457067	529841
**Singlets**	872548	1046891	1032868	477570	526394	685718
P. vulgaris/P. pseudovulgaris*	640791	715560	716878	470972	429492	417494
**Total**	1577596	1885375	1856562	886294	983461	1215559
	**Chr7**	**Chr8**	**Chr9**	**Chr10**	**Chr11**	**Not-mapped**
**Non-unique**	716128	718638	681648	369035	486946	706142
**Singlets**	916047	845538	875554	434598	577532	1432169
P. vulgaris/P. pseudovulgaris*	585065	663907	548797	401460	498863	-
**Total**	1632175	1564176	1557202	803633	1064478	2138311

*These SNPs present in *P. vulgaris* and *P. pseudovulgaris* were considered for the haplotype analyses in the domestication section.

Not-mapped column displays the number of SNPs called in contigs that were not assigned to any chromosomal position. These are mostly short contigs that do not reach the minimum number of CDSs to be placed in the pseudoassembly.

Table S12. Detected metabolites.

Name	m/z	Ionization	Exact Mass	Formula	RT	Ions
Trigonelline	138.05	[M+H]+	138.0555	C7H7NO2	0.33005	100 most abundant
Threonine	120.07	[M+H]+	120.0655	C4H9NO3	1.00735	100 most important
4-Methylumbelliferone	177.05	[M+H]+	177.0551	C10H8O3	2.236917	100 most important
D-Sorbitol 6-phosphate	263.05	[M+H]+	263.0532	C6H15O9P	0.3301	100 most important
Luteolin	287.04	[M+H]+	287.0555	C15H10O6	3.486033	100 most important
Kaempferol	287.04	[M+H]+	287.055	C15H10O6	3.295167	100 most important
Luteolin	287.04	[M+H]+	287.0555	C15H10O6	2.168317	100 most important
Kaempferol	287.04	[M+H]+	287.055	C15H10O6	5.202183	100 most important
Luteolin	287.04	[M+H]+	287.0555	C15H10O6	2.549283	100 most important
all-trans-Retinoic acid	301.20	[M+H]+	301.2167	C20H28O2	13.29615	100 most important
L-beta-Homotryptophan	219.09	[M+H]+	219.1133	C12H14N2O2	2.63535	100 most intense
Biotin	245.08	[M+H]+	245.0954	C10H16N2O3S	2.5326	100 most intense
Quercetin	303.03	[M+H]+	303.0504	C15H10O7	2.956717	100 most intense
Myricetin	319.03	[M+H]+	319.0449	C15H10O8	2.236917	100 most intense
alpha-Tocotrienol	425.37	[M+H]+	425.3419	C29H44O2	10.24525	100 most intense
Daidzin	439.12	[M+Na]+	439.0999	C21H20O9	0.3301	100 most intense
Kaempferol-7-neohesperidoside; LC-ESI-QTOF; MS2; [M+H]+; CE	595.15	[M+H]+	595.1658	C27H30O15	3.5719	100 most intense
Pelargonin	595.16	[M]+	595.1663	C27H31O15	2.168317	100 most intense
Isorhamnetin	317.06	[M+H]+	317.0656	C16H12O7	3.676733	30 most important
Coumarin	147.04	[M+H]+	147.0441	C9H6O2	2.549283	whole profile
D-Ala-D-ala	161.09	[M+H]+	161.0926	C6H12N2O3	2.635258	whole profile
Umbelliferone	163.03	[M+H]+	163.039	C9H6O3	14.83854	whole profile
DL-5-Hydroxylysine	163.10	[M+H]+	163.1082	C6H14N2O3	4.542092	whole profile
Nicotine	163.14	[M+H]+	163.1235	C10H14N2	11.59702	whole profile
Chalcone	209.11	[M+H]+	209.0966	C15H12O	2.253883	whole profile
gamma-Glutamylleucine	261.21	[M+H]+	261.2	C11H20N2O5	13.88493	whole profile
Genistein	271.05	[M+H]+	271.0601	C15H10O5	4.7327	whole profile
Cinchonine	295.18	[M+H]+	295.1805	C19H22N2O	14.45722	whole profile
Sinapoyl malate	341.09	[M+H]+	341.0872	C15H16O9	3.016733	whole profile
gamma-Tocotrienol	411.35	[M+H]+	411.3263	C28H42O2	17.04082	whole profile
Ononin	431.12	[M+H]+	431.1337	C22H22O9	3.016733	whole profile
Ideain	449.09	[M]+	449.1078	C21H21O11	2.549283	whole profile
Cyanidin-3-glucoside	449.10	[M]+	449.1084	C21H21O11	2.168317	whole profile
Kaempferol-3-Glucuronide	463.08	[M+H]+	463.0876	C21H18O12	3.31235	whole profile
Isoquercitrin	465.09	[M+H]+	465.1028	C21H20O12	2.046483	whole profile
Hyperoside	465.09	[M+H]+	465.1028	C21H20O12	3.016733	whole profile
Quercetin-3-Glucuronide	479.07	[M+H]+	479.0826	C21H18O13	2.91385	whole profile
Procyanidin B2	579.16	[M+H]+	579.1502	C30H26O12	2.618217	whole profile
Cyanidin-3-O-(2''-O-beta-xylopyranosyl-beta-glucopyranoside)	581.17	[M]+	581.1506	C26H29O15	3.016733	whole profile
Reserpine	609.26	[M+H]+	609.2806	C33H40N2O9	13.4009	whole profile
Cyanidin-3,5-di-O-glucoside	611.15	[M]+	611.1612	C27H31O16	2.168317	whole profile
Rutin	611.15	[M+H]+	611.1606	C27H30O16	3.016733	whole profile
Kaempferol-3-O-rutinoside	617.13	[M+Na]+	617.1477	C27H30O15	3.5719	whole profile
Quercetin-3-O-alpha-L-rhamnopyranosyl(1-2)-beta-D-glucopyranoside-7-O-alpha-L-rhamnopyranoside	757.21	[M+H]+	757.2191	C33H40O20	2.168317	whole profile

Table S20. Quantitative/morphological traits between *P. vulgaris* genotypes

**Characters**	**Mesoamerica**	**Amotape-Huancabamba**	**Andes**
Length of inflorescence peduncle (cm)	32 cm	1.0-4.0 cm	5.0 cm
Number of floral nodes per infl.	7-10	1-2	4-8
Number of veins on bracteoles	7-10	8	5-6
Flower colour	Lavender, pink or white	Pink	Lavender, pink-purple (not white)
No. of seeds/pod	8-10	5-8	5-8
Pod length (cm)	6-8.3	6-7.5	4.5-10.0
Pod width (cm)	0.5-1.0	0.8-0.9	0.6-1.0
100 seed weight (gr)	3.5-6.5	7.1-11	11.6-13.9
Seed length (mm)	6-10.0	7.0-8.0	5.0-10.0
Seed width (mm)	5-6	4.5-5.5	3.5-7.0
Hilum length (mm)	1.0	1.5 mm	1.5-1.8
