# Supplementary material for: Genomic history of the origin and domestication of common bean unveils its closest sister species
Source: Genome Biol. 2017 Mar 29;18:60. doi: 10.1186/s13059-017-1190-6 (PMC5370463; doi:10.1186/s13059-017-1190-6)
Supplement: Supplementary file 3 — Supplementary text, Tables S4–S11 and Figures S5–S10. Coalescent simulations. (PDF 693 kb) [file 13059_2017_1190_MOESM3_ESM.pdf]

Genomic history of the origin and domestication of common bean unveils its closest sister species

Additional file 3

Coalescent simulations

To obtain a temporal frame of the divergence between AH genotypes and the *P. vulgaris* clade, we conducted coalescent simulations using the Bayesian inference implemented in BEAST v.2.3.0 [68]. Three data sets from seven genotypes (P. vulgaris BAT93 and Jalo EEP558 as representative genotypes of the MA and Andean genepools, respectively; one accession from Peru (G21245), *P. dumosus*, *P. costaricensis*, *P. coccineus* and *P. hintonii*) were considered: 150 random protein coding genes (PCGs) from the 11 linkage groups; 170 widespread single-copy orthologous PCGs that were used by Valsova *et al*. 2016 to produce the phylogenetic tree of 13 plant species; 55Kb of the chloroplast genome. XML files for the simulations were produced with BEAUTi v.2.3.0 with the following priors: GTR substitution model with estimated frequencies; Yule birth tree model; log normal uncorrelated relaxed clock with adjusted clock rates according to the data set (Tables S4, 5); monophyly of *P. vulgaris* BAT93/Jalo EEP558 and monophyly of *P. dumosus*/*P. costaricensis.*

The XML files were fed as input to BEAST, to perform 3 MCMC runs with 10,000,000 steps (log every 1000). Log and tree files were combined with LogCombiner v.2.3.0; the consensus trees were obtained with TreeAnnotator v.2.3.0 and drawn with FigTree v.1.4.2 for each case. Consensus trees and Tracer statistics are displayed in Figures S5-10 and Tables S6-11.

Table S4. Time and μ priors considered for the chloroplast genome.

	Chloroplast genome			
**Priors**	**1**		**2**	
**Clade**	**Time (MY)**	**μ**	**Time (MY)**	**μ**
A	-	-	3	1.13E-3
Vulgaris (BAT93-JaloEEP558)	0.165	8.14e-4	-	-
Clock rate	-	8.14e-4	-	1.13E-3

Table S5. Time and μ priors considered for the nuclear markers.

	Random PCGs				Orthologous PCGs			
**Priors**	**1**		**2**		**3**		**4**	
**Clade**	**Time (MY)**	**μ**	**Time (MY)**	**μ**	**Time (MY)**	**μ**	**Time (MY)**	**μ**
A/B	1	1.75E-2	5	3.5E-03	1.6	1.2E-2	3.5	5.1E-3
A	0.7	1.1E-2	2.5	3.0E-03	1.8	4.7E-3	1.6	5.3E-3
Vulgaris (BAT93-JaloEEP558)	0.165	3.03E-04	0.165	3.03E-04	0.165	3.03E-04	0.165	3.03E-04
P. vulgaris/P. pseudovulgaris	-	-	0.7	6.0E-03	0.7	6.20E-03	0.7	6.20E-03
Clock rate	-	5.0E-3	-	3.50E-03	-	0.011	-	5.1E-3

Table S6. Statistics derived from the combination of three MCMC runs using the chloroplast markers (time priors [1], Table S4).

tMRCA	BAT93/Jalo	*P. vulgaris/ P.pseudovulgaris*	*P. dumosus/ P.costaricensis*	Clade A
**mean**	0.1711	0.9808	2.1804	3.8029
**stderr of mean**	2.7663E-3	0.0117	0.0101	0.0153
**stdev**	0.3108	0.8779	0.6211	1.1571
**variance**	0.0966	0.7706	0.3858	1.3389
**median**	0.1548	0.922	2.1338	3.7525
**geometric mean**	0.1563	0.9441	2.1477	3.7415
**95% HPD Interval**	[0.0663, 0.2887]	[0.582, 1.4424]	[1.5403, 2.9119]	[2.5401, 5.0339]
**auto-correlation time (ACT)**	2139.4536	4765.0878	7209.8729	4710.2285
**effective sample size (ESS)**	12620.9795	5666.6323	3745.1423	5732.6306

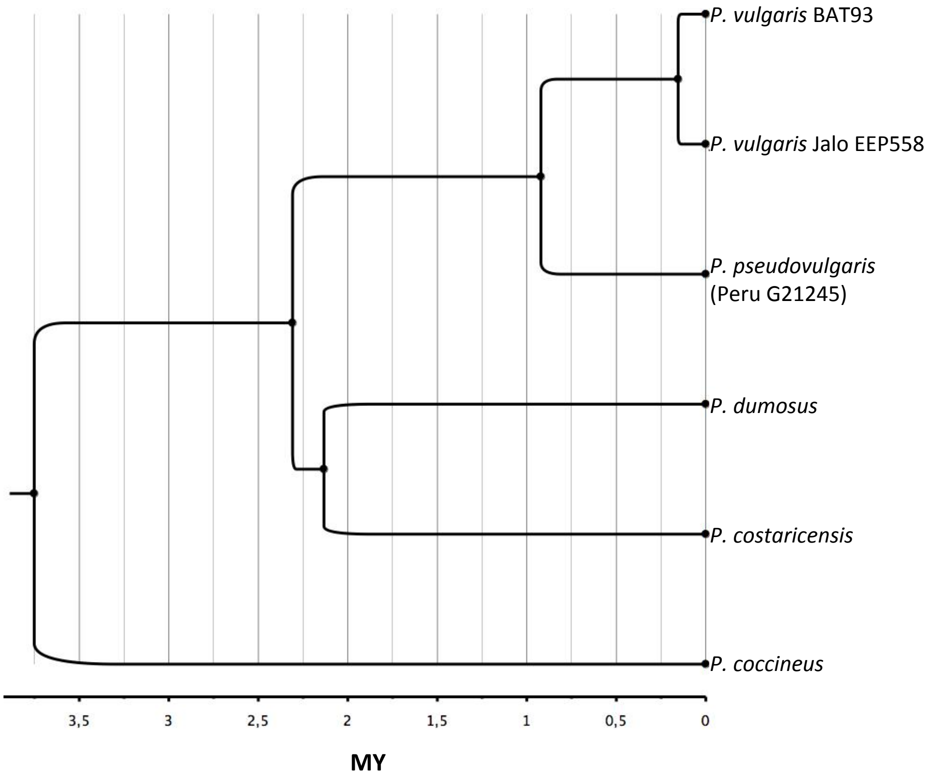

Figure S5. Consensus tree resulting from the combination of three MCMC runs using the chloroplast markers (time priors [1], Table S4).

Table S7. Statistics derived from the combination of three MCMC runs using the chloroplast markers (time priors [2], Table S4).

**tMRCA**	**BAT93/Jalo**	***P. vulgaris/ P.pseudovulgaris***	***P. dumosus/ P.costaricensis***	**Clade A**
**mean**	0.1269	0.7021	1.5656	2.731
**stderr of mean**	7.2413E-3	0.0114	4.7861E-3	0.0114
**stdev**	1.1871	1.5387	0.4613	1.497
**variance**	1.4092	2.3676	0.2128	2.2411
**median**	0.1112	0.6613	1.5318	2.6993
**geometric mean**	0.1119	0.6725	1.5438	2.6841
**95% HPD Interval**	[0.0484, 0.2062]	[0.423, 1.0038]	[1.0849, 2.0854]	[1.8173, 3.6215]
**auto-correlation time (ACT)**	1004.7639	1494.6592	2906.9868	1556.4576
**effective sample size (ESS)**	26873.9738	18065.6569	9288.6559	17348.3689

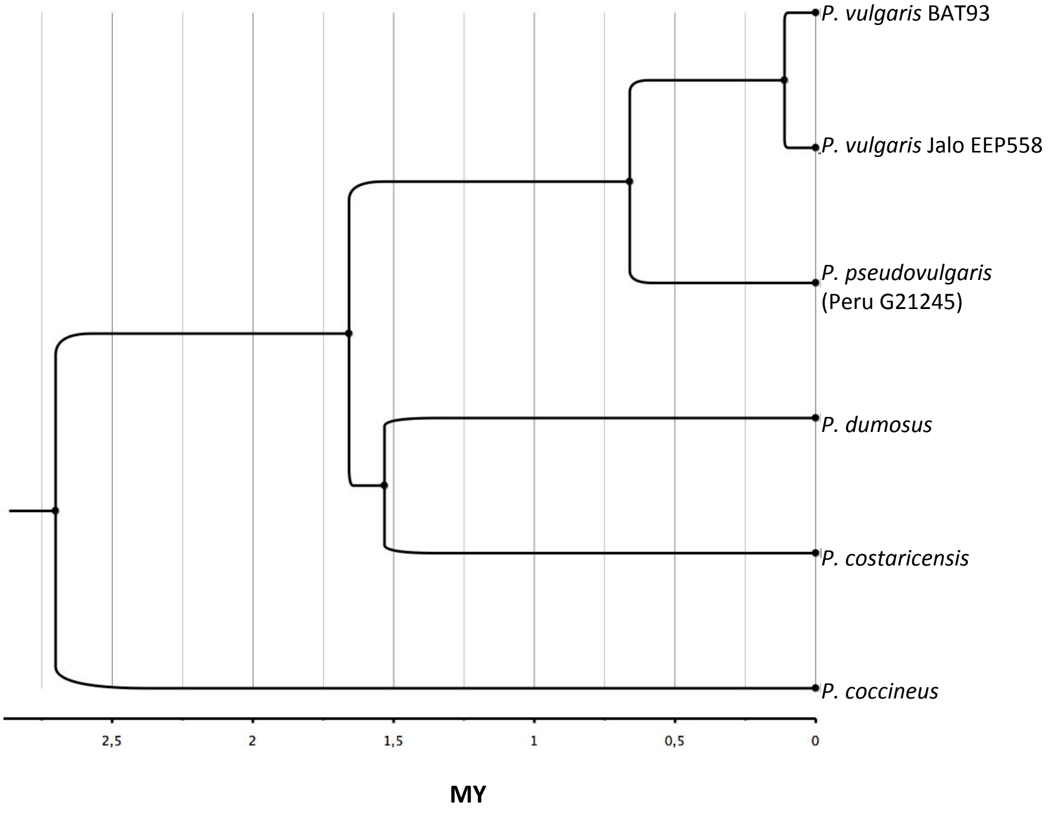

Figure S6. Consensus tree resulting from the combination of three MCMC runs using the chloroplast markers (time priors [2], Table S4).

Table S8. Statistics derived from the combination of three MCMC runs using the nuclear gene set of 150 random PCGs (time priors [1], Table S5).

tMRCA	BAT93/Jalo	*P. vulgaris/ P.pseudovulgaris*	P. dumosus/ P.costaricensis	Clade A	Clades A/B
**mean**	2.2794E-3	0.2571	0.1302	0.4513	0.9691
**stderr of mean**	1.1064E-4	6.6727E-3	1.6422E-3	4.1744E-3	0.0332
**stdev**	9.8066E-3	0.2218	0.1126	0.3775	0.8387
**variance**	9.617E-5	0.0492	0.0127	0.1425	0.7033
**median**	1.6321E-3	0.1953	0.0981	0.3452	0.7324
**geometric mean**	1.6343E-3	0.1935	0.0979	0.344	0.7278
**95% HPD Interval**	[1.4643E-4, 5.9619E-3]	[0.0181, 0.6753]	[6.8602E-3, 0.3404]	[0.0356, 1.1651]	[0.067, 2.5336]
**auto-correlation time (ACT)**	3436.9495	24446.7783	5741.4606	3302.2315	42269.8842
**effective sample size (ESS)**	7856.6763	1104.5627	4703.1587	8177.1977	638.8236

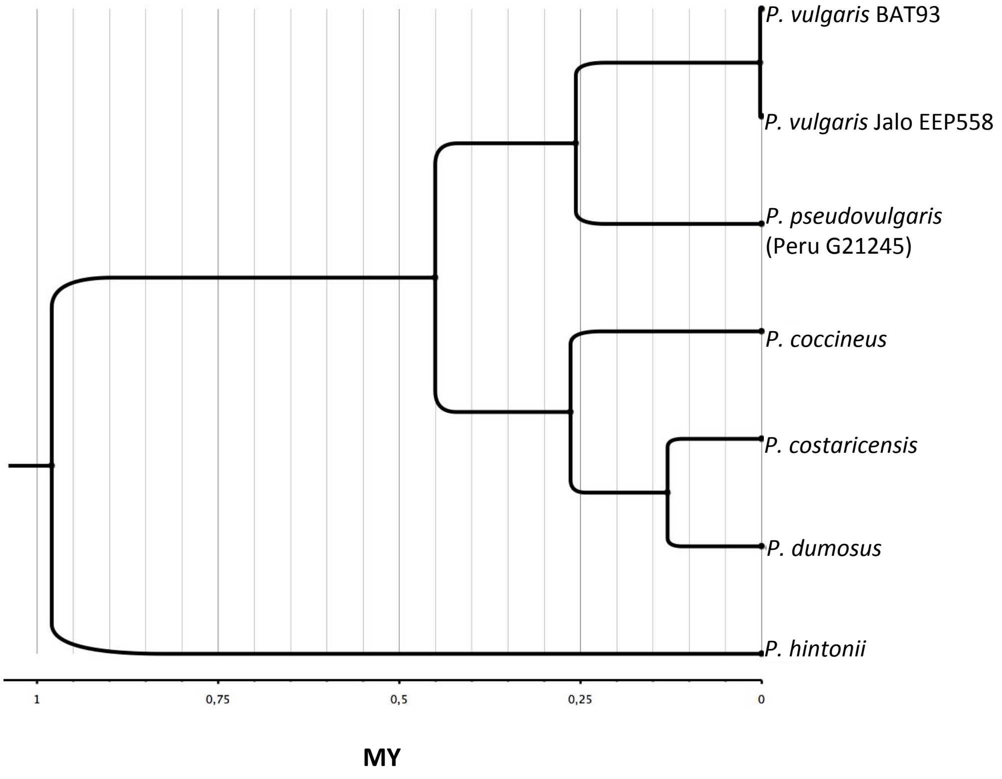

Figure S7. Consensus tree resulting from the combination of three MCMC runs using nuclear gene set of 150 random PCGs (time priors [1], Table S5).

Table S9. Statistics derived from the combination of three MCMC runs with the nuclear gene set of 150 random PCGs (time priors [2], Table S5).

tMRCA	BAT93/Jalo	*P. vulgaris/ P.pseudovulgaris*	*P. dumosus/ P.costaricensis*	Clade A	Clades A/B
**mean**	1.8725E-3	0.244	0.121	0.4259	0.9982
**stderr of mean**	2.8544E-5	3.0918E-3	1.4402E-3	2.77E-3	0.0102
**stdev**	4.1243E-3	0.174	0.0875	0.2982	0.7043
**variance**	1.701E-5	0.0303	7.6479E-3	0.0889	0.496
**median**	1.4802E-3	0.1987	0.098	0.3481	0.8164
**geometric mean**	1.4833E-3	0.1989	0.0982	0.3491	0.8164
**95% HPD Interval**	[2.1633E-4, 4.4264E-3]	[0.0309, 0.5773]	[0.0157, 0.2866]	[0.0614, 0.9997]	[0.1357, 2.353]
**auto-correlation time (ACT)**	1293.435	8524.293	7323.528	2330.6788	5624.228
**effective sample size (ESS)**	20876.9667	3167.77	3687.1574	11585.8951	4801.1923

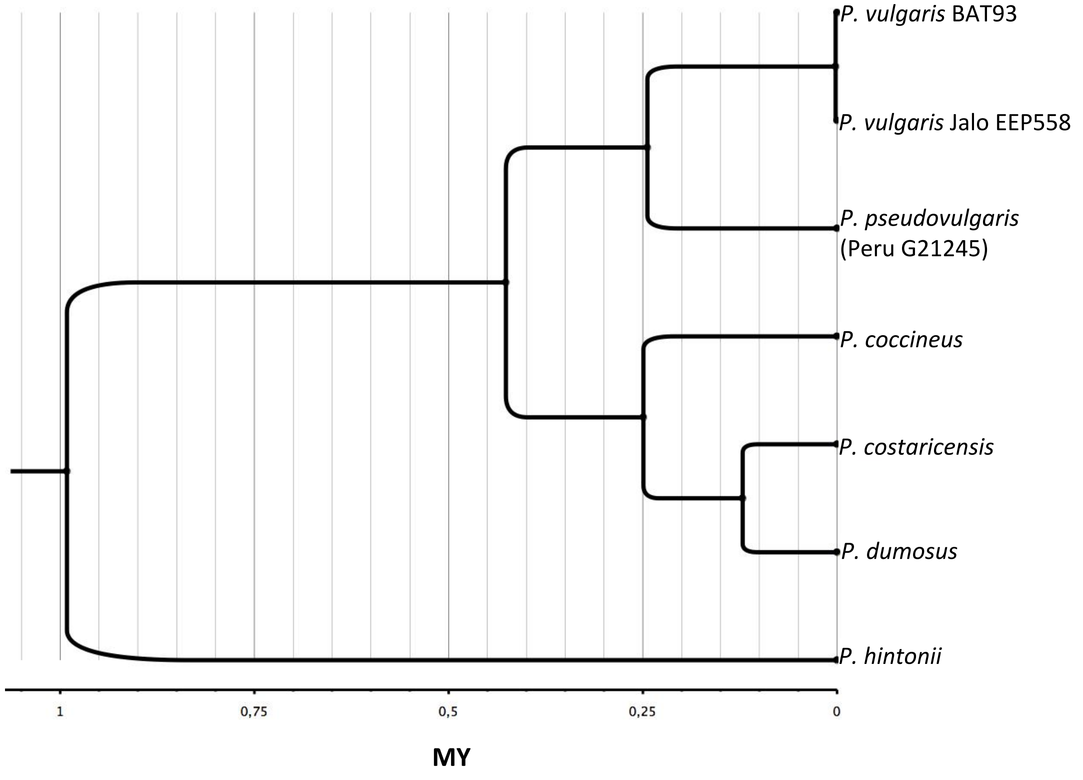

Figure S8. Consensus tree resulting from the combination of three MCMC runs using nuclear gene set of 150 random PCGs (time priors [2], Table S5).

Table S10. Statistics derived from the combination of three MCMC runs using the nuclear gene set of 170 orthologous PCGs (time priors [3], Table S5).

tMRCA	BAT93/Jalo	*P. vulgaris/ P.pseudovulgaris*	*P. dumosus/ P.costaricensis*	Clade A	Clades A/B
**mean**	2.0333E-3	0.2337	0.1352	0.4335	0.9672
**stderr of mean**	5.2135E-5	0.0108	6.1489E-3	2.7912E-3	0.0236
**stdev**	8.1384E-3	0.1682	0.099	0.3016	0.6813
**variance**	6.6234E-5	0.0283	9.7987E-3	0.091	0.4642
**median**	1.6089E-3	0.1905	0.1093	0.3554	0.7909
**geometric mean**	1.605E-3	0.1894	0.109	0.3551	0.7893
**95% HPD Interval**	[2.2032E-4, 4.723E-3]	[0.0312, 0.5632]	[0.0144, 0.3233]	[0.0534, 1.0088]	[0.1171, 2.2651]
**auto-correlation time (ACT)**	1108.155	1.1047E5	1.042E5	2312.4709	32483.8748
**effective sample size (ESS)**	24367.5305	244.4283	259.1577	11677.12	831.274

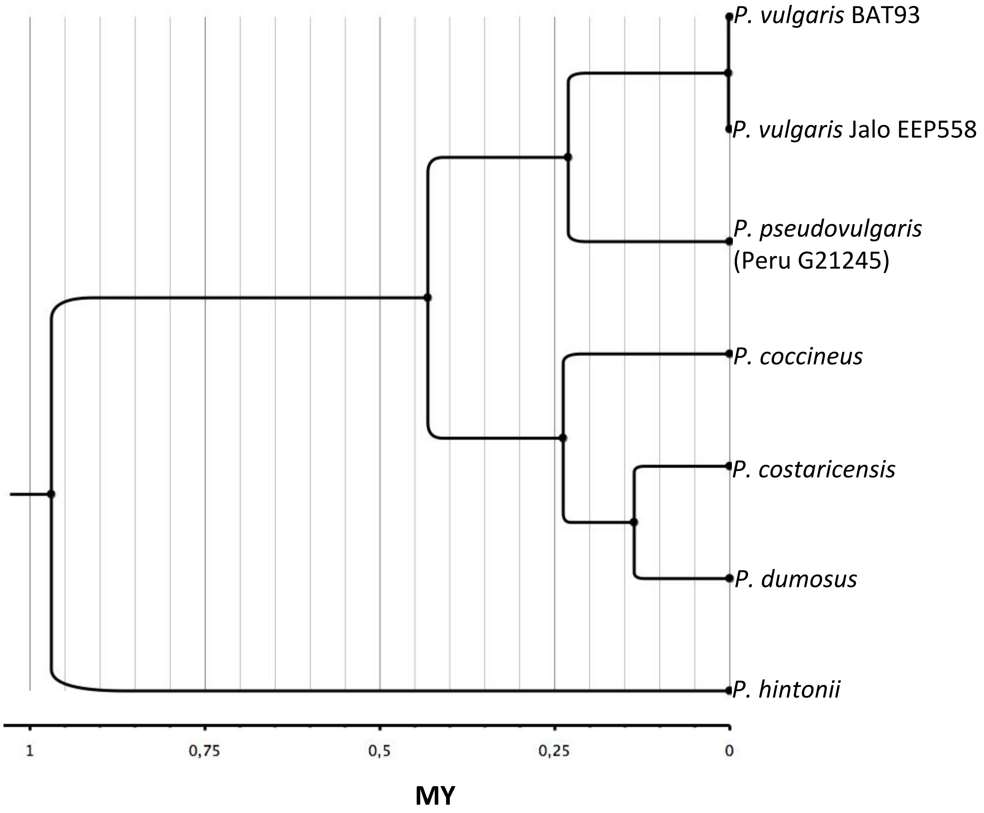

Figure S9. Consensus tree resulting from the combination of three MCMC runs using nuclear gene set of 170 orthologous PCGs (time priors [3], Table S5).

Table S11. Statistics derived from the combination of three MCMC runs with the nuclear gene set of 150 random PCGs (time priors [4], Table S5).

tMRCA	BAT93/Jalo	*P. vulgaris/ P.pseudovulgaris*	*P. dumosus/ P.costaricensis*	Clade A	Clades A/B
**mean**	2.0593E-3	0.2265	0.145	0.4372	0.9987
**stderr of mean**	2.3018E-5	4.7058E-3	3.1918E-3	3.3402E-3	0.053
**stdev**	2.2591E-3	0.1613	0.1103	0.3082	0.7233
**variance**	5.1037E-6	0.026	0.0122	0.095	0.5231
**median**	1.6545E-3	0.1843	0.1176	0.3594	0.806
**geometric mean**	1.6545E-3	0.1844	0.1175	0.3578	0.8074
**95% HPD Interval**	[2.2953E-4, 4.9063E-3]	[0.0267, 0.5329]	[0.018, 0.3442]	[0.0517, 1.0233]	[0.1274, 2.3816]
**auto-correlation time (ACT)**	2803.2992	22975.8216	22599.1929	3171.9187	1.4486E5
**effective sample size (ESS)**	9632.5787	1175.279	1194.8657	8513.1439	186.4043

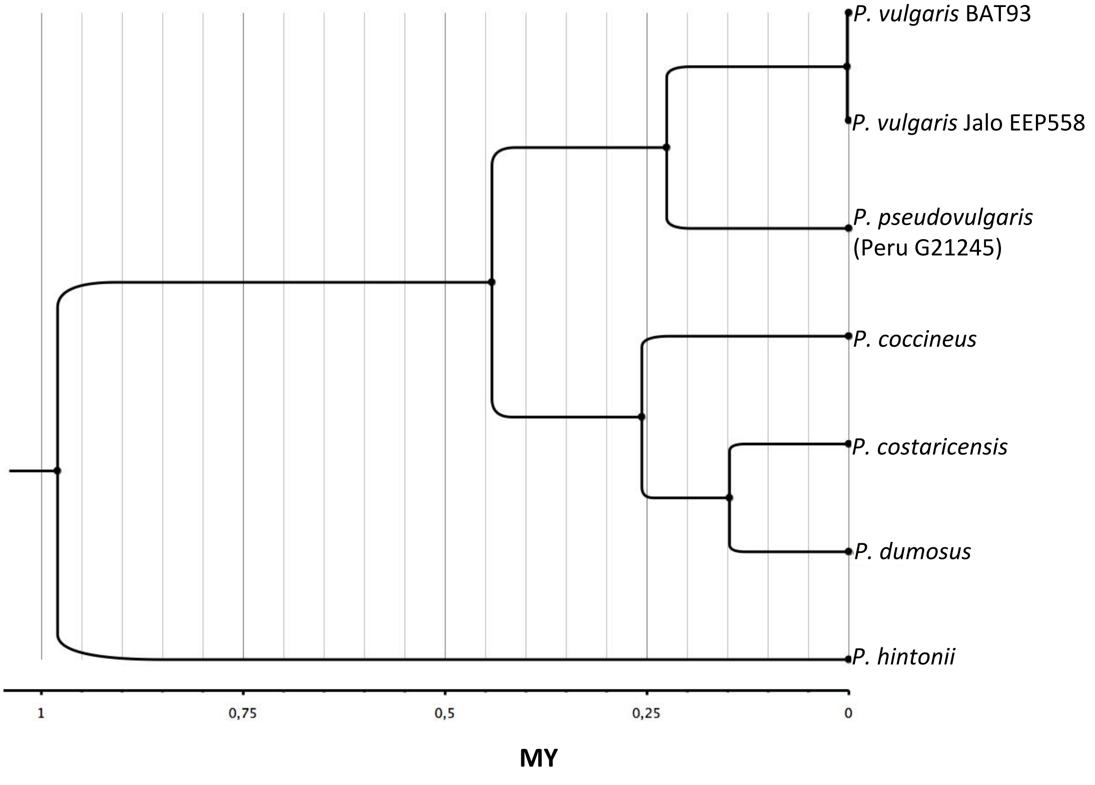

Figure S10. Consensus tree resulting from the combination of three MCMC runs using nuclear gene set of 170 orthologous PCGs (time priors [4], Table S5).
